# A newly developed method for assessing co-exposure to free dose combinations: a proof-of-concept study using antihypertensive medications in Danish registers

**DOI:** 10.1007/s40520-024-02879-4

**Published:** 2024-11-26

**Authors:** Maria Antonietta Barbieri, Sidse Marie Neumann Nielsen, Andrea Rossi, Elena Olmastroni, Manuela Casula, Edoardo Spina, Maurizio Sessa

**Affiliations:** 1 Department of Drug Design and Pharmacology, University of Copenhagen, Jagtvej 160, 2100 Copenhagen, Capital Region Denmark; 2 Department of Clinical and Experimental Medicine, University of Messina, 98125 Messina, Italy; 3 Epidemiology and Preventive Pharmacology Service (SEFAP), Department of Pharmacological and Biomolecular Sciences, University of Milan, Milan, Italy; 4 IRCCS MultiMedica, Sesto S. Giovanni, Milan Italy

**Keywords:** Antihypertensive medications, Electronic health records databases, Free dose combination, Hypertension, Register-based cohort study

## Abstract

**Background:**

Elevated blood pressure is a major risk factor for severe medical conditions. Adherence to antihypertensive medication, especially in free-dose combinations, poses a significant challenge. This study aims to develop a novel method for assessing co-exposure to free-dose antihypertensive medications using secondary data sources.

**Methods:**

A register-based cohort study was conducted on individuals aged 65 years or older in Denmark who initiated antihypertensive therapy from 1996 to 2016 and followed for 730 days from the index date. A new method was developed to assess co-exposure to antihypertensive medications through redeemed prescriptions, treatment episodes, and overlapping medication events. The method's accuracy was evaluated using a random sample of 400 individuals.

**Results:**

A total of 1,021,819 individuals were included in the study, with a mean age of 68.8 years, and 53.7% were women. The method achieved 100% accuracy in identifying co-exposure periods. During the early stage of the follow-up (0–180 days), 54.1% of individuals were co-exposed to at least two antihypertensive medications, while 37.5% were co-exposed during the late stage of the follow-up period (181–730 days). The most frequent antihypertensive combinations included bendroflumethiazide and potassium with either amlodipine or enalapril in the early (13.2% and 12.5% of patients, respectively) and late stages (16.9% and 15.0% of patients, respectively).

**Conclusions:**

The newly developed method effectively assesses co-exposure to antihypertensive medications, overcoming previous limitations. The findings reveal common co-exposure combinations and evolving trends in antihypertensive medication use among older individuals, reflecting changes in clinical practice and guidelines over two decades.

**Supplementary Information:**

The online version contains supplementary material available at 10.1007/s40520-024-02879-4.

## Introduction

Elevated blood pressure is a major risk factor for severe medical conditions, including chronic kidney disease and diabetes, all of which rank among the leading global causes of death [1]. Various global, international, and national committees recommend combination therapy with free-dose or fixed-dose antihypertensive medication in their hypertension treatment guidelines [2]. Free-dose combinations involve prescribing and dispensing two or more separate antihypertensives medications taken individually by the patient. In contrast, fixed-dose combinations are single pills containing a predetermined combination of antihypertensive medications in fixed dosages [3]. Adherence [4] is a major challenge in hypertension treatment especially among individuals treated with free dose combinations [5]. The prevalence of nonadherence to antihypertensive treatment varies across studies due to differences in assessment methods, healthcare systems, and patient populations [6, 7]. In large population samples, approximately one-third of hypertensive patients are nonadherent, with newly treated patients being particularly prone to non-persistence [8]. The prevalence of antihypertensive medication nonadherence to free dose combinations was estimated to be between 27 and 40% depending on the detection method used in a study from 2022 [5]. Nonadherence is strongly correlated with higher blood pressure and adverse cardiovascular outcomes leading to increased healthcare costs and mortality [8]. The high prevalence of nonadherence among users of free dose combinations contributes to poor blood pressure control, leading to severe clinical and economic consequences especially among older individuals [5, 9, 10]. When dealing with antihypertensive medications, it is often necessary to use methods that are able to measure adherence indirectly from secondary data sources [11]. Based on a systematic review of the literature recently conducted, several methods have been developed to assess adherence to free dose antihypertensive combination therapy in various ways. While these methods have provided valuable insights, they are universally limited by a key challenge: the inability to accurately assess co-exposure to two or more antihypertensive medications taken concurrently (Online Research 1). Indeed, after analyzing twelve relevant studies, we identified this limitation as a significant gap in existing research, with none of the methods providing a robust solution to accurately assess co-exposure to multiple antihypertensive medications. To address this gap, this study has two aims: first, to develop a new method for assessing co-exposure to antihypertensive free-dose combination therapy in secondary data sources; second, to apply the method to describe co-exposure to selected antihypertensive medications among older individuals in Denmark who initiated treatment with a free-dose combination during the first two years of treatment in the period 1996–2018.

## Methods

### Data sources

Data for the study was available during the study period from the 1st of January 1995 to the 31st of December 2018 and were collected from the Danish Civil Registration System [12], the Danish Register of Causes of Death [13], the Register of Medicinal Product Statistics [14], the Danish Income Statistics Register [15], the Danish Population Education Register [16], and the Danish National Patient Register [17].

### Study design and study population

This register-based cohort study comprised individuals aged 65 years or older for at least one day during the study period. The study population included individuals initiating therapy with diuretics (*Anatomical Therapeutic Chemical Classification System*, ATC codes: C03AA), calcium channel blockers, CCBs (ATC: C08CA), angiotensin-converting enzyme inhibitors, ACEi (ATC: C09AA), and/or angiotensin II receptor blockers, ARBs (ATC: C09CA) at the index date, from January 1, 1996, to December 31, 2016. The index date, defined as the initiation of antihypertensive therapy (index medication redemption), served as the starting point for participant enrollment, commencing from January 1, 1996. A one-year period preceding the index date was designated for washout purposes to ensure the inclusion of new users. The study design is outlined in Online Research Fig. S1.

### Follow-up

The study population was followed for 730 days from the index date. Individuals were censored upon completion of the 730-day follow-up period, emigration, or death from any cause. The follow-up period was further divided into an early stage of treatment (180 days) and a later stage (181–730 days) to investigate co-exposure to antihypertensive medications separately in these two stages.

### Outcome

The primary study outcome was the accuracy of individuals correctly classified by the newly developed method as being in co-exposure to two or more combinations of antihypertensive medications. The secondary study outcome focused on the top five most redeemed combinations of antihypertensive medications in Denmark during the study period.

### New method

The proposed method initiates by selecting redeemed prescriptions for antihypertensive medications within the study population during the follow-up period. Then, the method proceeds with three additional steps:

1. Assessment of the duration of medication events during the follow-up period and construction of treatment episodes separately for each antihypertensive medication;
2. Assessment of co-exposure to antihypertensive medications;
3. Creation of a final treatment episode of co-exposure to at least two free dose and/or fixed dose combinations of antihypertensive medications for each individual in the study population.

The method has been developed in R and the code is provided in Online Research 2.

#### Step 1

**Fig. 1:**
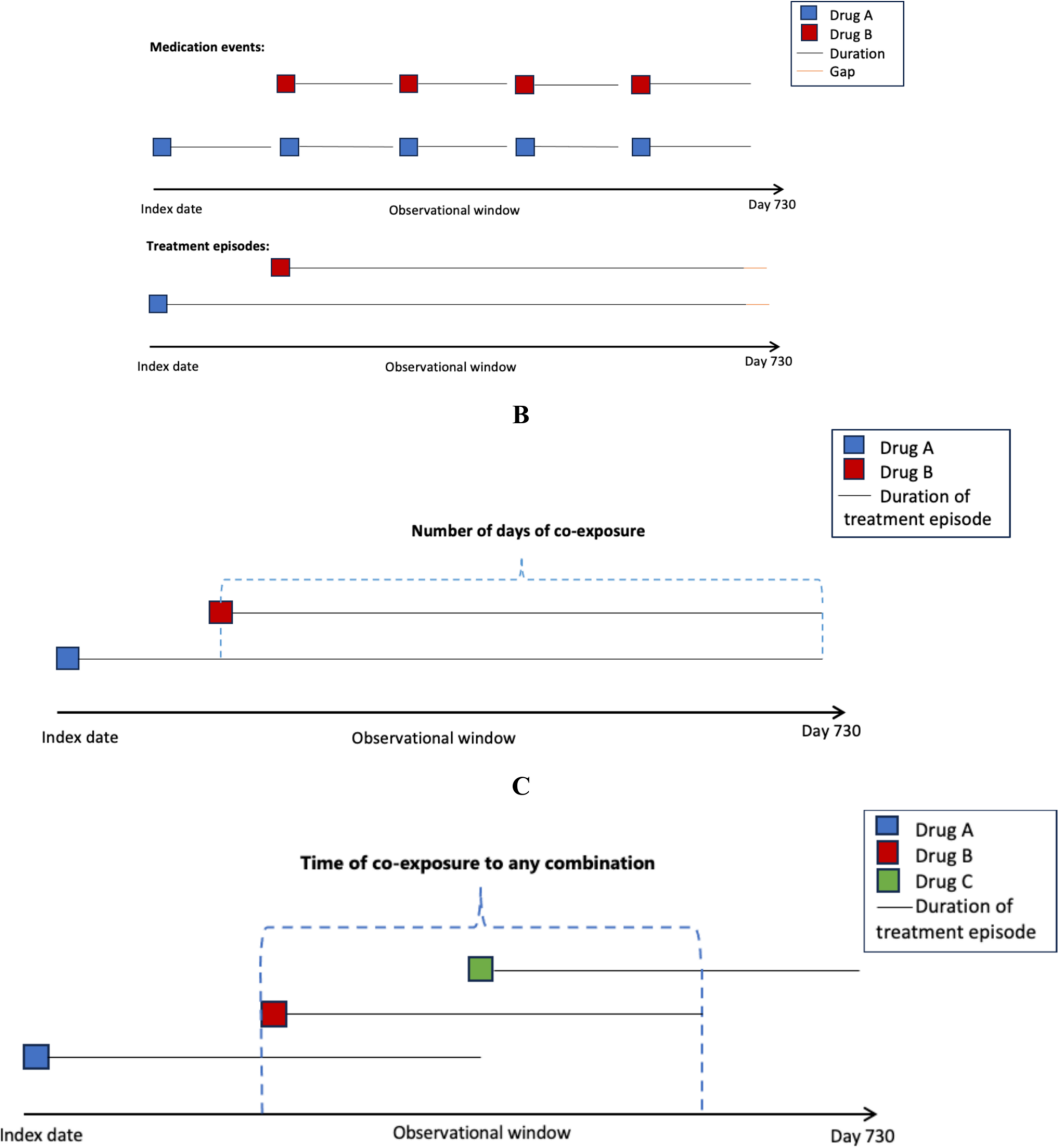
The newly developed method: Step 1 (panel **A**), Step 2 (panel **B**), and Step 3 (panel **C**)

We estimated the duration of medication events for antihypertensive medications in the study population using the Sessa Empirical Estimator method, based on the articles by Meaidi and Pazzagli [18–20]. Next, we identified treatment episodes for each antihypertensive medication using the *compute.treatment.episodes* function from the AdhereR package [21]. Treatment episodes were generated using carry-over only for the same medication, with a maximum permissible gap of 30 days [22] at the end of the last medication event in each episode (Fig. 1—panel A). If carry-over resulted in the number of days with available medication exceeding the length of the follow-up period, the treatment episode was capped at 730 days.

#### Step 2

We assessed the duration of co-exposure for each combination of free-dose antihypertensive medications by calculating the number of days in overlapping treatment episodes using the functions described in Online Research 2. In cases with multiple overlapping episodes, the total co-exposure duration was the sum of these overlapping periods. Co-exposure for fixed-dose combinations was not calculated using the same approach because the duration estimated with the Sessa Empirical Estimator is already considered the number of days of co-exposure for the specific fixed-dose combinations (Fig. 1—panel B).

#### Step 3

We used the function *compute.treatment.episodes* from the AdhereR package to construct a final treatment episode (Fig. 1—panel C) summarizing the total number of days of co-exposure to two or more free-dose and/or fixed-dose antihypertensive medication combinations during the follow-up period. This episode excludes carry-over and does not allow for any gaps in medication use.

#### Additional functions

To identify overlapping periods of co-exposure to multiple medication pairs for each patient, we designed additional functions that utilize data generated in Step 2. The *check_overlap* function determines if two periods overlap by comparing their start and end dates. The *find_overlaps* function iterates through each patient's medication pair data, using *check_overlap* to identify overlapping co-exposure periods to multiple pairs. The *combine_pairs* function merges and sorts the medication pairs, and this new information is added to the data frame.

### Statistical analysis

Baseline characteristics of the study population were analyzed to provide an overview of the study population. This included calculating the frequency distribution of the first redeemed antihypertensive medication, sex (male/female), mean age, income level, and highest level of education attained for the study population.

To evaluate the method's performance, we selected a random sample of 400 individuals from the study population. Performance was measured by the percentage of correctly classified start and end dates of co-exposure to two or more antihypertensive medications. We conducted an individual evaluation of the start and end date of the co-exposure period for each patient in this sample by plotting all their medication events. Online Research 3 provides technical details for this procedure, along with an example using simulated data.

The top five combinations of antihypertensive medications redeemed in Denmark during the study period (1996–2018) were presented in timeline diagrams for four-year groups (1996–2000, 2001–2005, 2006–2010, 2011–2015, and 2016–2018). These diagrams depict usage patterns by active substance during the early (0–180 days) and later (181–730 days) stages of treatment. Statistical analyses were conducted using R (Vienna, R Core Team 2024) [23].

### Ethics

Regional ethics committee approval was not required under Danish law [24]. Individual-level data was stored only at Statistics Denmark. All analyses were performed on aggregated data that did not identify any individual. Data handling followed the guidelines of Directive 95/46/EC (General Data Protection Regulation) and the Danish Data Protection Act (Databeskyttelsesloven). All results presented in this study include at least five observations. This study was reported according to recently approved standards for reporting pharmacoepidemiological studies (i.e., STROBE and HARPER) [25, 26].

## Results

### Study population

A total of 1,021,819 individuals aged 65 or above who redeemed an antihypertensive medication between 1996 and 2016 were included in the study (Table 1). The mean age was 68.8 years, with women comprising a higher proportion (53.7%). The initial prescribed antihypertensives included diuretics (thiazides with and without potassium), ACEi, CCBs, and ARBs. Specifically, the antihypertensive medications were bendroflumethiazide and potassium (43.7%), amlodipine (15.5%), enalapril (13.7%), ramipril (7.4%), and losartan (5.6%). The study population was followed for an average of 701 days, with 68,531 individuals (6.9%) censored. Among the study population, 360,266 individuals (35.5%) redeemed more than one antihypertensive medication during the follow-up period.

**Table 1:** Baseline characteristics of the study population

Variable	Patient (*n* = 1,021,819)
First antihypertensive medication, *n* (%)	
Bendroflumethiazide and potassium	432,866 (43.7)
Amlodipine	153,033 (15.5)
Enalapril	135,815 (13.7)
Ramipril	72,898 (7.4)
Losartan	55,774 (5.6)
Perindopril	22,358 (2.3)
Trandolapril	21,675 (2.2)
Candesartan	16,050 (1.6)
Felodipine	14,537 (1.5)
Lisinopril	11,918 (1.2)
Nifedipine	11,628 (1.2)
Captopril	75,42 (0.8)
Irbesartan	6946 (0.7)
Bendroflumethiazide	6834 (0.7)
Valsartan	5628 (0.6)
Telmisartan	3806 (0.4)
Lercanidipine	1668 (0.2)
Fosinopril	1475 (0.1)
Eprosartan	1227 (0.1)
Hydroflumethiazide and potassium	1147 (0.1)
Quinapril	1115 (0.1)
Lacidipine	912 (0.1)
Isradipine	785 (0.1)
Olmesartan medoxomil	641 (0.1)
Mibefradil	282 (< 0.1)
Nitrendipine	265 (< 0.1)
Hydrochlorothiazide	218 (< 0.1)
Nilvadipine	217 (< 0.1)
Moexipril	207 (< 0.1)
Nimodipine	185 (< 0.1)
Benazepril	133 (< 0.1)
Sex (male) *n* (%)	458,498 (46.3)
Age (year), mean (SD)	68.8 (10.4)
Equivalized household income (Danish kroner, DKK), mean (SD)	174,402.3 (227,054.3)
Highest achieved education, *n* (%)	
No education	857 (0.1)
Compulsory school and 10th grade	400,284 (46.6)
Vocational education and training and adult education	16,628 (1.9)
Upper secondary certificate (Gymnasium)	11,340 (1.3)
Academic profession degrees	280,089 (32.6)
Bachelor and diploma degrees	131,464 (15.3)
Candidatus and master degrees	12,762 (1.5)
PhD	1572 (0.2)
Other educations and/or unspecified educations	4340 (0.5)

*SD* standard deviation, *PhD* Doctor of Philosophy

### Performance of the newly developed method

The newly developed method achieved perfect accuracy (100%) in identifying co-exposure to antihypertensive medications and correctly classifying the start and end dates of the co-exposure period for patients redeeming more than one antihypertensive medication at the same time.

### Co-exposure to antihypertensive medications

**Fig. 2:**
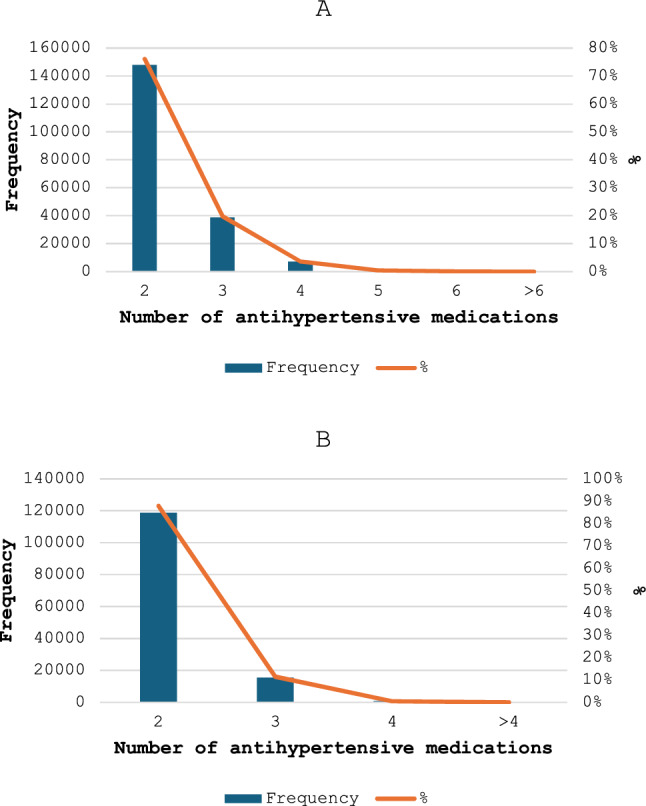
Number of combinations the individual patients were co-exposed to: Early stage (panel **A**); and late stage (panel **B**)

In all, 194,599 older individuals (54.1%) had a co-exposure to at least two antihypertensive medications in the early stage, while 135,111 (37.5%) had a combination to at least two antihypertensives medications during the late stage. Figure 2 depicts the most commonly co-exposed combinations of antihypertensive medications during the follow-up period. Specifically, during the early stage, 148,032 individuals (76.1%) were co-exposed to two antihypertensive medications, 38,618 (19.8%) to three antihypertensives, and 7059 (3.6%) to four antihypertensives (Fig. 2—panel A). During the late stage, 118,825 (87.9%) individuals were co-exposed to two antihypertensive medications, and 15,525 (11.5%) to three or more medications (Fig. 2—panel B).

#### Most commonly redeemed combinations of antihypertensive medications: early stage

In the early stages of follow-up, the most frequent co-exposures to two antihypertensive medications involved the combination of bendroflumethiazide and potassium with either amlodipine or enalapril, with an incidence of 13.2% and 12.5% of patients, respectively, among individuals receiving two antihypertensive medications during the early stage of the follow-up period. These combinations had median durations of 99 days. Other frequently observed incident co-exposures included amlodipine with enalapril and bendroflumethiazide and potassium with ramipril (Table 2—panel A). Although less common, combinations such as bendroflumethiazide and potassium with losartan, and amlodipine with losartan, still accounted for around 3% of patients. The duration of these co-exposures generally ranged from 88 to 100 days, indicating varied treatment lengths (Table 2—panel A). In the early stages of follow-up, the most frequent triple therapy involved bendroflumethiazide and potassium with both amlodipine and enalapril, with an incidence of 2.6% of patients, with a median duration of 119 days. Another common regimen was bendroflumethiazide and potassium combined with amlodipine and ramipril, with an incidence of 1.5% of the cohort and had a longer median duration of 129 days. Other combinations, such as those involving losartan, perindopril, or trandolapril, were observed in less than 1% of patients each but typically exceeded 115 days in median duration (Table 2—panel B).

**Table 2:** Top 20-incident co-exposure to two (panel A) or more than two (panel B) antihypertensive medications in the early stage of follow-up

Co-exposure	Patient (*n* = 194,599)	Median duration (Q1–Q3)
A		
Bendroflumethiazide and potassium + amlodipine	25,761 (13.2)	99 (49–152)
Bendroflumethiazide and potassium + enalapril	24,389 (12.5)	99 (51–150)
Amlodipine + enalapril	11,658 (6.0)	86 (33–134)
Bendroflumethiazide and potassium + ramipril	9334 (4.8)	100 (55–151)
Amlodipine + ramipril	7128 (3.7)	96 (46–149)
Bendroflumethiazide and potassium + losartan	6270 (3.2)	92 (42–142)
Amlodipine + losartan	5877 (3.0)	88 (35–138)
Bendroflumethiazide and potassium + perindopril	3821 (2.0)	101 (50–154)
Bendroflumethiazide and potassium + trandolapril	3761 (1.9)	101 (55–156)
Bendroflumethiazide and potassium + felodipine	3455 (1.8)	97 (45–151)
Bendroflumethiazide and potassium + candesartan	3211 (1.7)	94 (44–145)
Bendroflumethiazide and potassium + bendroflumethiazide	1667 (0.9)	60 (26–94)
Bendroflumethiazide and potassium + lisinopril	1641 (0.8)	91 (42–139)
Amlodipine + trandolapril	1602 (0.8)	95 (38–152)
Amlodipine + perindopril	1549 (0.8)	98 (39–156)
Bendroflumethiazide and potassium + irbesartan	1151 (0.6)	93 (43–141)
Amlodipine + candesartan	1125 (0.6)	79 (28–126)
Bendroflumethiazide and potassium + captopril	1068 (0.6)	93 (53–139)
Felodipine + enalapril	1068 (0.6)	78 (29–123)
Amlodipine + lisinopril	1005 (0.5)	85 (34–130)
B		
Bendroflumethiazide and potassium + amlodipine + enalapril	5140 (2.6)	119 (84–163)
Bendroflumethiazide and potassium + amlodipine + ramipril	2834 (1.5)	129 (97–172)
Bendroflumethiazide and potassium + enalapril and diuretics + enalapril	1826 (0.9)	129 (106–158)
Bendroflumethiazide and potassium + amlodipine + losartan	1748 (0.9)	117 (82–160)
Bendroflumethiazide and potassium + amlodipine + perindopril	887 (0.5)	131 (99–175)
Bendroflumethiazide and potassium + amlodipine + trandolapril	809 (0.4)	126 (90–171)
Bendroflumethiazide and potassium + felodipine + enalapril	666 (0.3)	118 (82–160)
Bendroflumethiazide and potassium + amlodipine + candesartan	629 (0.3)	118 (78–165)
Amlodipine + losartan + ramipril	557 (0.3)	99 (62–136)
Bendroflumethiazide and potassium + amlodipine + lisinopril	424 (0.2)	114 (76–160)
Bendroflumethiazide and potassium + amlodipine + losartan and diuretics	320 (0.2)	124 (87–165)
Bendroflumethiazide and potassium + felodipine + ramipril	258 (0.1)	124 (90–167)
Bendroflumethiazide and potassium + amlodipine + enalapril and diuretics	242 (0.1)	115 (77–154)
Bendroflumethiazide and potassium + felodipine + losartan	209 (0.1)	116 (82–160)
Bendroflumethiazide and potassium + amlodipine + irbesartan	178 (0.1)	110 (67–159)
Bendroflumethiazide and potassium + amlodipine + valsartan	159 (0.1)	117 (85–158)
Bendroflumethiazide and potassium + amlodipine + captopril	150 (0.1)	124 (89–169)
Bendroflumethiazide + amlodipine + enalapril	129 (0.1)	113 (77–160)
Bendroflumethiazide and potassium + nifedipine + enalapril	128 (0.1)	115 (79–160)
Bendroflumethiazide and potassium + felodipine + perindopril	100 (0.1)	123 (83–171)

#### Most commonly redeemed combinations of antihypertensive medications: late stage

In the late stage of follow-up, co-exposures to two antihypertensive medications frequently involved the combination of bendroflumethiazide and potassium with either amlodipine or enalapril, with an incidence of 16.9% and 15.0%, respectively, each having a median duration of 292 days. Other combinations included amlodipine with enalapril and bendroflumethiazide and potassium with ramipril, with an incidence of 6.5% and 6.0%, respectively. These regimens also displayed extended median durations of 292 and 289 days, respectively (Table 3—panel A). For triple or more antihypertensive medication co-exposure, the most frequent regimen in the late stage involved bendroflumethiazide and potassium combined with both amlodipine and enalapril, with an incidence of 2.5% of patients, and a median duration of 367 days. Other common triple combinations included bendroflumethiazide and potassium and amlodipine with either ramipril or losartan, covering 1.7% and 1.2% of the cohort respectively, each with median durations exceeding 346 days (Table 3—panel B).

**Table 3:** Top 20-incident co-exposure to two (panel A) or more than two (panel B) antihypertensive medications in the late stage of follow-up

Co-exposure	Patient (*n* = 135,111)	Median duration (Q1–Q3)
A		
Bendroflumethiazide and potassium + amlodipine	22,812 (16.9)	292 (153–432)
Bendroflumethiazide and potassium + enalapril	20,246 (15.0)	292 (159–429)
Amlodipine + enalapril	8828 (6.5)	292 (153–429)
Bendroflumethiazide and potassium + ramipril	8171 (6.0)	289 (154–427)
Bendroflumethiazide and potassium + losartan	7883 (5.8)	265 (132–394)
Amlodipine + losartan	6635 (4.9)	276 (143–406)
Amlodipine + ramipril	6071 (4.5)	301 (170–439)
Bendroflumethiazide and potassium + candesartan	4478 (3.3)	266 (140–392)
Bendroflumethiazide and potassium + perindopril	3367 (2.5)	282 (143–423)
Bendroflumethiazide and potassium + felodipine	3162 (2.3)	296 (156–434)
Bendroflumethiazide and potassium + trandolapril	3071 (2.3)	278 (138–410)
Amlodipine + perindopril	1432 (1.1)	309 (175–448)
Amlodipine + candesartan	1423 (1.1)	267 (135–395)
Amlodipine + trandolapril	1360 (1.0)	279 (130–420)
Bendroflumethiazide and potassium + irbesartan	1341 (1.0)	270 (132–409)
Bendroflumethiazide and potassium + lisinopril	1301 (1.0)	280 (142–418)
Bendroflumethiazide and potassium + valsartan	1147 (0.8)	262 (129–384)
Bendroflumethiazide and potassium + telmisartan	876 (0.6)	270 (134–396)
Bendroflumethiazide and potassium + captopril	826 (0.6)	261 (129–394)
Felodipine + enalapril	824 (0.6)	281 (140–420)
B		
Bendroflumethiazide and potassium + amlodipine + enalapril	3316 (2.5)	367 (278–469)
Bendroflumethiazide and potassium + amlodipine + ramipril	2305 (1.7)	375 (288–475)
Bendroflumethiazide and potassium + amlodipine + losartan	1685 (1.2)	346 (250–453)
Bendroflumethiazide and potassium + amlodipine + perindopril	737 (0.5)	374 (274–483)
Bendroflumethiazide and potassium + amlodipine + candesartan	701 (0.5)	360 (273–462)
Bendroflumethiazide and potassium + amlodipine + trandolapril	606 (0.4)	370 (277–480.8)
Bendroflumethiazide and potassium + felodipine + enalapril	528 (0.4)	372 (281–475)
Bendroflumethiazide and potassium + amlodipine + lisinopril	249 (0.2)	350 (270–457)
Bendroflumethiazide and potassium + felodipine + ramipril	237 (0.2)	370 (283–473)
Bendroflumethiazide and potassium + amlodipine + irbesartan	200 (0.1)	338 (251–439)
Bendroflumethiazide and potassium + felodipine + losartan	199 (0.1)	352 (276–451)
Bendroflumethiazide and potassium + amlodipine + valsartan	190 (0.1)	348 (263–453)
Bendroflumethiazide and potassium + candesartan + enalapril	140 (0.1)	273 (182–362)
Bendroflumethiazide and potassium + amlodipine + telmisartan	136 (0.1)	342 (248–447)
Bendroflumethiazide and potassium + felodipine + candesartan	122 (0.1)	362 (292–450)
Bendroflumethiazide and potassium + felodipine + perindopril	98 (0.1)	356 (260–467)
Bendroflumethiazide and potassium + amlodipine + captopril	97 ()	359 (260–451)
Bendroflumethiazide and potassium + lercanidipine + enalapril	94 ()	341 (253–439)
Bendroflumethiazide and potassium + nifedipine + enalapril	91 ()	367 (292–459)
Bendroflumethiazide + amlodipine + enalapril	90 ()	328 (222–434)

#### Variation over time in the top five antihypertensive medication combinations during early and late stages

**Fig. 3:**
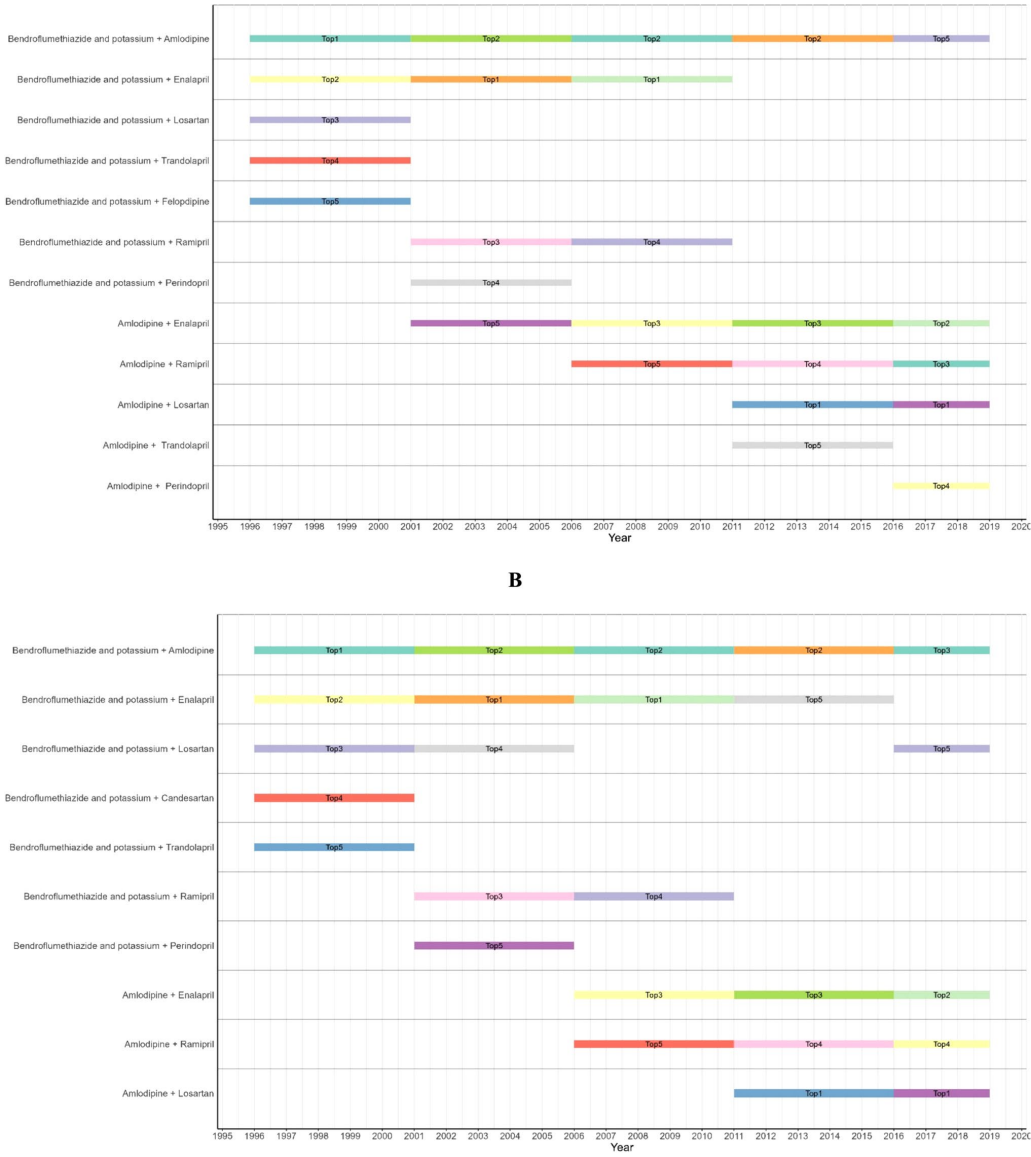
Variation over years of the top five combinations of antihypertensive medications during initial period (index date-day 180) (panel **A**) and late period (day 181–730) (panel **B**)

Figure 3—Panel A shows the variation over the years of the top five combinations of antihypertensive medications in the early stage. Some of these combinations maintained their top positions for extended periods. For example, the co-exposure to bendroflumethiazide and potassium with amlodipine consistently appeared as Top 1 or Top 2 from 1996 to 2016, indicating its sustained prominence. Other combinations, such as bendroflumethiazide and potassium with enalapril, showed long-term usage as Top 1 and Top 2 until 2012, then giving way to increasingly used combinations in recent years amlodipine with losartan, which has been the Top 1 since 2012. Moreover, amlodipine with enalapril had extended and increased usage in the later years, moving from Top 5 in 2001–2005 to Top 2 in the last two years.

In the late stage, Fig. 3—Panel B shows a similar variation in the top five medication combinations. The combination of bendroflumethiazide and potassium with amlodipine again featured prominently, maintaining top positions throughout most of the observed years, underscoring its continued importance. Notable shifts have occurred with bendroflumethiazide and potassium in combination with enalapril, which fluctuated among the top ranks since 2012, due to the presence of new combinations such as amlodipine with enalapril and amlodipine with losartan in later years, reflecting evolving practices in hypertension management in late stages.

## Discussion

This study aimed to develop a new method for assessing exposure to free-dose antihypertensive medications using Danish registers. Antihypertensives were chosen as the proof-of-concept medication group in this study due to the high global prevalence of hypertension [27] and the need for using multiple antihypertensive medications simultaneously in clinical practice [28, 29]. The newly developed method was applied to a study population of 1,021,819 new users of antihypertensives aged 65 years or older in Denmark. These prevalence estimates of new users of antihypertensives aged 65 years or older in Denmark align with those provided by the Danish Heart Foundation (Hjerteforeningen) [30]. The study population was followed for an extensive period, with a mean follow-up of 701 days. At the 4th level ATC-code, the most commonly prescribed medications at the index date in the study population were diuretics (i.e., thiazides) with and without potassium, followed by ACEi, CCBs, and ARBs. The choice of initially prescribed medications aligns with recommendations from clinical guidelines, as these drug classes are all considered first-line agents [2, 31, 32] and are supported by another Danish study [33]. Indeed, the active substances bendroflumethiazide and potassium, as well as amlodipine are the first choice for the treatment of hypertension according to Danish guidelines [34].

The mean age of the study population was approximately 69 years. Despite the inclusion of older adults in this study, it is important to acknowledge that patients starting antihypertensive therapy are often younger, representing a different demographic. However, the study population resembles similar cohorts in other countries, underlining its relevance as a reflection of the general older population [35]. The study cohort consisted predominantly of women (approximately 54%). Given that hypertension prevalence tends to be higher in men, it was anticipated that new users above 65 years old were more likely to be women, as men are expected to initiate treatment at an earlier age [36].

Our method achieved perfect performance in assessing co-exposure to multiple antihypertensive medications, overcoming the limitations found in other methods for evaluating co-exposure to combination therapy of antihypertensive medications mentioned in Online Research 1. Bias and pharmacoepidemiological flaws directly or indirectly affected these previous methods. Firstly, the newly developed method did not compromise on transparency, as all the key operational procedures to compute the duration of medication events and assess co-exposure were clearly described. The method has been detailed, and the code for the function in R has been attached in Online Research 2 for reproducibility. Immortal time bias was avoided by recruiting all new users of an antihypertensive medication and aligning the follow-up start at the first redeemed prescription of an antihypertensive medication, thereby not conditioning on a future event (i.e., the start of combination therapy of antihypertensive medications) [37].

Notably, a substantial proportion of individuals aged 65 and above redeemed more than one antihypertensive medication during the follow-up period. Specifically, more than half of these patients had co-exposure to at least two antihypertensive medications in the early stage period, and 37.5% in the late stage period of the follow-up period. This reflects a complex treatment landscape aimed at effectively managing hypertension while considering individual patient profiles and treatment responses. The study identified specific combinations of antihypertensive medications that were commonly co-exposed during the early and late stages of follow-up. Specifically, combinations involving bendroflumethiazide and potassium with amlodipine or enalapril were prominent across both stages, indicating their sustained effectiveness and possibly favorable side effect profiles in older populations [38–40]. Moreover, some individuals were exposed to more than two antihypertensive combinations simultaneously. Longer durations in accordance with the median duration for the late stage and the co-exposure to three or more antihypertensive medications suggest a more intensive management approach for patients requiring multiple medications. The study's findings also highlight temporal trends in the use of specific antihypertensive combinations over the study period. For instance, the combination of bendroflumethiazide and potassium with amlodipine consistently ranked among the top combinations, reflecting its enduring popularity and perceived clinical effectiveness [39, 40]. Newer combinations like amlodipine with losartan gained prominence in later years, suggesting shifts in clinical practice and treatment guidelines favoring newer agents or combinations with potentially different effectiveness or safety profiles. The observed variability in these combinations over time underscores evolving clinical guidelines and therapeutic preferences in hypertension management [34, 40]. This shift might also be explained by the ONTARGET trial (Telmisartan, Ramipril, or Both in Patients at High Risk for Vascular Events) in 2008, which demonstrated that ARBs are an equally effective alternative to ACEi with a better safety profile [40]. Additionally, in 2009, the patent for losartan expired, which can explain the shift from ACEi to ARBs in the combination treatment regimens [40].

One of the key take-home messages from our study, especially when examining the most prescribed antihypertensive drugs combinations, is the emerging shift from free-dose combinations to fixed-dose combinations, particularly in the context of dual antihypertensive single-dose therapy. This shift highlights the increasing awareness among healthcare providers of the benefits of fixed-dose combinations in managing hypertension. Fixed-dose combinations have demonstrated a significant impact on improving adherence patterns, which is clearly reflected in our results, as the preference of healthcare providers seems to shift towards these fixed combinations. As treatment regimens become more complex with the need for multiple medications, especially in hypertension management, it is crucial to emphasize the importance of simplifying treatment regimens whenever possible. Simplified regimens, such as fixed dose combinations, can help enhance adherence and optimize patient outcomes [34, 40].

This study has several limitations that should be considered in interpreting the findings. The observational design may introduce selection bias, as the study population may not accurately represent the global demographic of older adults with hypertension, and prescribing practices can vary widely across countries. While the newly developed method successfully identified co-exposure to antihypertensive medications, potential misclassifications may arise from errors in prescription records. Although the focus was on co-exposure during the follow-up period, we could not account for changes in medication regimens thereafter. The study’s reliance on redeemed prescriptions does not confirm actual medication intake, potentially leading to discrepancies between prescribed and consumed medications. Additionally, while we identified commonly co-exposed medication combinations, the analysis did not consider the influence of underlying comorbidities, lifestyle factors, or concomitant medications, all of which can significantly affect treatment decisions and outcomes. Lastly, the temporal trends observed may be impacted by shifts in clinical guidelines, the introduction of new medications, or changes in healthcare practices.

## Conclusion

This study developed a novel method for assessing exposure to free-dose antihypertensive medications using Danish registers, focusing on a cohort of older individuals initiating treatment. The method demonstrated robustness and transparency, addressing some limitations found in previous approaches. Through meticulous analysis, the study revealed notable trends in antihypertensive medication combinations over time among older individuals in Denmark, providing insights into changing prescription patterns and potential influencing factors, such as guidelines, clinical trials, and patent expirations. By identifying prevalent combinations and temporal trends in medication use, this research highlights the evolving nature of hypertension management strategies over the past two decades. Looking forward, the study sets a precedent for future research directions in pharmacoepidemiology and clinical practice. Continued monitoring of medication patterns, guided by updated clinical guidelines and emerging therapeutic options, will be important in optimizing cardiovascular outcomes and minimizing adverse effects in older populations. Additionally, further investigation into the long-term effectiveness and safety of newer antihypertensive combinations, including ARBs like losartan, is recommended. As hypertension remains a significant public health challenge globally, ongoing research efforts should focus on personalized medicine approaches. By addressing these areas, researchers and clinicians can work towards improving hypertension management and overall cardiovascular health in older adults.

## Supplementary Information

Below is the link to the electronic supplementary material.

Supplementary file1 (DOCX 2332 KB)

## Data Availability

Data are stored on secure servers on Statistics Denmark. Access can be granted by Statistics Denmark upon adequate permissions.
