# Supplementary material for: A newly developed method for assessing co-exposure to free dose combinations: a proof-of-concept study using antihypertensive medications in Danish registers
Source: Aging Clin Exp Res. 2024 Nov 26;36(1):226. doi: 10.1007/s40520-024-02879-4 (PMC11599442; doi:10.1007/s40520-024-02879-4)
Supplement: Supplementary file 1 — Supplementary file1 (DOCX 2332 KB) [file 40520_2024_2879_MOESM1_ESM.docx]

**Online Research**


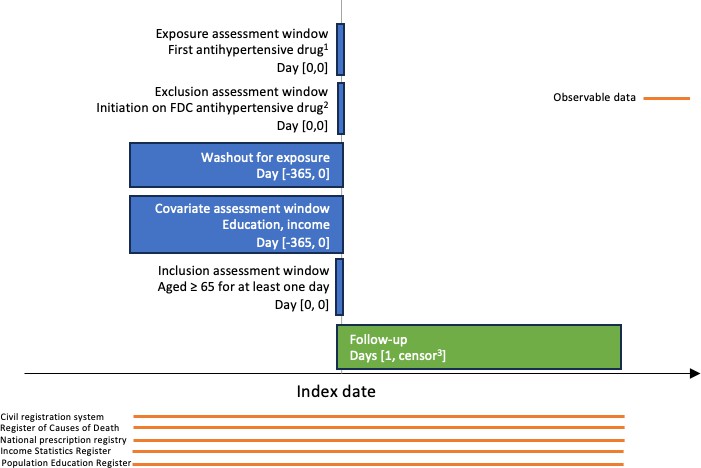


**Fig. S1.** Study design diagram.

1Antihypertensive drugs: diuretics (ATC: C03AA), calcium channel blockers (ATC: C08CA), angiotensin converting enzyme inhibitors (ATC: C09AA), or angiotensin II blockers (ATC: C09CA)

2 Fixed-dose combination (FDC) antihypertensive drugs: angiotensin converting enzyme inhibitors and diuretics (ATC: C09BA),

angiotensin converting enzyme inhibitors and calcium channel blockers (ATC: C09BB), angiotensin II blockers and diuretics (C09DA), angiotensin II blockers and calcium channel blockers (ATC: C09DB), and angiotensin II blockers and others (ATC: C09DX).

3 Censoring: (1) the end of the follow-up period (i.e., day 730), (2) emigration, or (3) death from any cause during the follow-up.

**Online Research 1. Systematic review**

***Online Research 1.1 Method of systematic review***

The literature database PubMed was used to search the structured research query on the 1st of September 2023. The National Center for Biotechnology Information (NCBI) maintains PubMed, and its primary component is MEDLINE, which is the premier bibliographic database of the National Library of Medicine (NLM) [1].

The search query (Online Research, Table 1) was constructed using four blocks separated by AND. Keyword synonyms for the search terms "adherence" (Block 1) and "hypertension" (Block 2) were generated using reviews by Nieuwlaat et al. [2] and Arguedas et al. [3], respectively, from the Cochrane Database of Systematic Reviews. Keywords within the blocks were combined using OR. The search period (1st of January 1950 to 31st of August 2023) was defined in block 3, and block 4 excluded Clinical Trials publications. The filters "Full text available," "English," and "Humans" were applied. The systematic review was conducted according to the Preferred Reporting Items for Systematic Reviews and Meta-Analyses (PRISMA) guidelines [4]. Observational studies that developed methods for co-exposure assessment to free dose antihypertensives were evaluated. Therefore, studies that did not consider free dose combination therapy were excluded. Additionally, articles with unrelated content and studies that did not use secondary data sources for adherence measurement (e.g., questionnaires, self-reports, etc.) were excluded. Only original research was considered eligible; thus, meta-analyses, systematic reviews, letters to the editor/researcher, commentary, and podcasts were excluded. Only articles with full texts available in English were eligible, and duplicates were excluded. In the first stage, articles were screened by their title and abstract. All articles considered eligible, and articles where it was not possible to assess the eligibility criteria from the abstract underwent full-text evaluation in the second stage.

**Online Research 1, Table 1.** MEDLINE Research query performed on 01/09/2023

| **Blocks** | **Query** | **N. of articles** |
| --- | --- | --- |
| Final research block | (((("1950/01/01"[Date - Entry] : "2023/08/31"[Date - Entry])) AND (((((("Hypertension"[Mesh]) OR (hypertension)) OR (hypertens*)) OR ("Blood Pressure"[Mesh])) OR (blood pressure)) OR (bloodpressure))) AND ((((("Patient Compliance"[Mesh]) OR (patient compliance)) OR (patient adherence)) OR (medication compliance)) OR (medication adherence))) NOT (Clinical trial[Publication Type])  /  1950/01/01:2023/08/31[Date - Entry] AND ("Patient Compliance"[MeSH Terms] OR ("Patient Compliance"[MeSH Terms] OR ("patient"[All Fields] AND "compliance"[All Fields]) OR "Patient Compliance"[All Fields]) OR ("Patient Compliance"[MeSH Terms] OR ("patient"[All Fields] AND "compliance"[All Fields]) OR "Patient Compliance"[All Fields] OR ("patient"[All Fields] AND "adherence"[All Fields]) OR "patient adherence"[All Fields]) OR ("medication adherence"[MeSH Terms] OR ("medication"[All Fields] AND "adherence"[All Fields]) OR "medication adherence"[All Fields] OR ("medication"[All Fields] AND "compliance"[All Fields]) OR "medication compliance"[All Fields]) OR ("medication adherence"[MeSH Terms] OR ("medication"[All Fields] AND "adherence"[All Fields]) OR "medication adherence"[All Fields])) AND ("Hypertension"[MeSH Terms] OR ("hypertense"[All Fields] OR "Hypertension"[MeSH Terms] OR "Hypertension"[All Fields] OR "hypertension s"[All Fields] OR "hypertensions"[All Fields] OR "hypertensive"[All Fields] OR "hypertensive s"[All Fields] OR "hypertensives"[All Fields]) OR "hypertens*"[All Fields] OR "Blood Pressure"[MeSH Terms] OR ("Blood Pressure"[MeSH Terms] OR ("blood"[All Fields] AND "pressure"[All Fields]) OR "Blood Pressure"[All Fields] OR "blood pressure determination"[MeSH Terms] OR ("blood"[All Fields] AND "pressure"[All Fields] AND "determination"[All Fields]) OR "blood pressure determination"[All Fields] OR ("blood"[All Fields] AND "pressure"[All Fields]) OR "Blood Pressure"[All Fields] OR "arterial pressure"[MeSH Terms] OR ("arterial"[All Fields] AND "pressure"[All Fields]) OR "arterial pressure"[All Fields] OR ("blood"[All Fields] AND "pressure"[All Fields])) OR "bloodpressure"[All Fields]) | 7,885 |
| Block 4 | NOT (Clinical trial[Publication Type]) |  |
| Block 3 - Period | ("1950/01/01"[Date - Entry] : "2023/08/31"[Date - Entry]) |  |
| Block 2 - Hypertension | ((((("Hypertension"[Mesh]) OR (hypertension)) OR (hypertens*)) OR ("Blood Pressure"[Mesh])) OR (blood pressure)) OR (bloodpressure)  /  "Hypertension"[MeSH Terms] OR ("hypertense"[All Fields] OR "Hypertension"[MeSH Terms] OR  "Hypertension"[All Fields] OR "hypertension s"[All Fields] OR "hypertensions"[All Fields] OR "hypertensive"[All Fields] OR "hypertensive s"[All Fields] OR "hypertensives"[All Fields]) OR "hypertens*"[All Fields] OR "Blood Pressure"[MeSH Terms] OR ("Blood Pressure"[MeSH Terms] OR ("blood"[All Fields] AND "pressure"[All Fields]) OR "Blood Pressure"[All Fields] OR "blood pressure determination"[MeSH Terms] OR ("blood"[All Fields] AND "pressure"[All Fields] AND "determination"[All Fields]) OR "blood pressure determination"[All Fields] OR ("blood"[All Fields] AND "pressure"[All Fields]) OR "Blood Pressure"[All Fields] OR "arterial pressure"[MeSH Terms] OR ("arterial"[All Fields] AND "pressure"[All Fields]) OR "arterial pressure"[All Fields] OR ("blood"[All Fields] AND "pressure"[All Fields])) OR "bloodpressure"[All Fields] | 1,103,917 |
| Block 1 - Adherence | (((("Patient Compliance"[Mesh]) OR (patient compliance)) OR (patient adherence)) OR (medication compliance)) OR (medication adherence)  **/**  "Patient Compliance"[MeSH Terms] OR ("Patient Compliance"[MeSH Terms] OR ("patient"[All Fields] AND "compliance"[All Fields]) OR "Patient Compliance"[All Fields]) OR ("Patient Compliance"[MeSH Terms] OR ("patient"[All Fields] AND "compliance"[All Fields]) OR "Patient Compliance"[All Fields] OR ("patient"[All Fields] AND "adherence"[All Fields]) OR "patient adherence"[All Fields]) OR ("medication adherence"[MeSH Terms] OR ("medication"[All Fields] AND "adherence"[All Fields]) OR "medication adherence"[All Fields] OR ("medication"[All Fields] AND "compliance"[All Fields]) OR "medication compliance"[All Fields]) OR ("medication adherence"[MeSH Terms] OR ("medication"[All Fields] AND "adherence"[All Fields]) OR "medication adherence"[All Fields]) | 179,423 |

***Online Research 1.2 Results of systematic review***

From the search in PubMed with the search string a total of 7885 articles were identified. The screening procedure and selection of included studies are presented using the PRISMA flowchart (Online Research 1, Fig. 1) from Moher et al. [4].


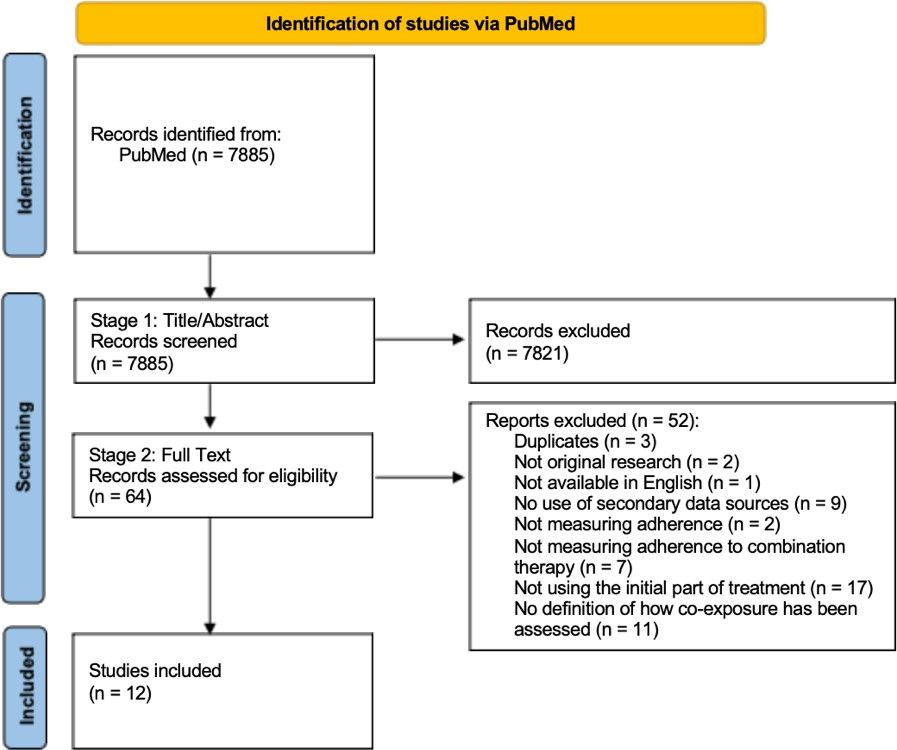


**Online Research 1, Fig. 1.** PRISMA Flowchart. Fig. adapted from Moher et al. [4]

In the preliminary stage 1 screening of titles and abstracts, 7,821 articles did not meet the inclusion criteria and were excluded, leaving 64 articles for stage 2 screening with full-text reading. At this stage, a total of 52 articles were excluded for reasons including duplicates (n = 3), not being original research such as commentary or review (n = 2), and not being available in English (n = 1). Additionally, 9 articles were excluded for not using secondary data sources for adherence measures. Exclusion was applied if articles did not measure adherence at all or did not measure adherence for combination therapy, resulting in the exclusion of 2 and 7 articles, respectively. Articles that did not measure adherence based on the initial part of the treatment were deemed ineligible, leading to the exclusion of 17 articles. Lastly, articles were excluded if the authors did not define how they assessed co-exposure, excluding 11 articles and leaving a total of 12 articles to be included (Online Research 1, Table 2). All the included articles have been published within the last 9 years, from 2015 to 2022. In the following pages, a summary of the methods and the main limitations of the studies is provided.

**Online Research 1, Table 2**. Studies included from systematic review.

| **Number** | **Title** | **Reference** |
| --- | --- | --- |
| 1 | Evaluating Adherence to Concomitant Diabetes, Hypertension, and Hyperlipidemia Treatments and Intermediate Outcomes Among Elderly Patients Using Marginal Structural Modeling | Paranjpe R. [5] |
| 2 | Non-persistence to antihypertensive drug therapy in Lithuania | Treciokiene I. [6] |
| 3 | Persistence and adherence to antihypertensive drugs in newly treated hypertensive patients according to initial prescription | Jeong S.M. [7] |
| 4 | Effect of patient and treatment factors on persistence with antihypertensive treatment: A population-based study | Malo S. [8] |
| 5 | Adherence to Treatment by Initial Antihypertensive Mono and Combination Therapies | Rea F. [9] |
| 6 | Antihypertensive Medication Nonpersistence and Low Adherence for Adults <65 Years Initiating Treatment in 2007-2014. | Tajeu G.S. [10] |
| 7 | Drug adherence in treatment resistant and in controlled hypertension-Results from the Swedish Primary Care Cardiovascular Database (SPCCD) | Holmqvist L. [11] |
| 8 | Associations among medication regimen complexity, medical specialty, and medication possession ratio in newly diagnosed hypertensive patients: A population-based study | Ho C.P. [12] |
| 9 | Antihypertensive Medication Adherence and Risk of Cardiovascular Disease Among Older Adults: A Population‐Based Cohort Study | Yang Q. [13] |
| 10 | Measuring medication adherence in patients with incident hypertension: a retrospective cohort study | Tang K. [14] |
| 11 | Patient Adherence to Olmesartan/Amlodipine Combinations: Fixed Versus Extemporaneous Combinations | Levi M. [15] |
| 12 | Comparison of amlodipine/valsartan/hydrochlorothiazide single pill combination and free combination: adherence, persistence, healthcare utilization and costs | Machnicki G. [16] |

**Article 1: Paranjpe et al., 2022**

In the article by Paranjpe et al. [5] the authors proposed a method to assess adherence to concomitant statins, oral antidiabetics, and renin-angiotensin-system (RAS) antagonists (combination therapy). In this study, patients on concurrent combination therapy were identified in the period June 2016 - December 2016, which was defined as identification period. Only patients that had at least one prescription of *“1) oral antidiabetic 2) statins and 3) RAS antagonists with at least one month of overlap of all three drugs in the identification period”* were included in the study population. The follow-up period was from January 2017 to December 2017 [5].

As illustrated in Online Research 1, Fig. 2, medication adherence was assessed every six months starting from the index date. The authors used the proportion of days covered (PDC) to measure adherence, and *“considered patients adherent to concurrent triple therapy if they had 80% or more days covered for any RAS antagonist, any statin, and any oral antidiabetic during the follow-up period”* (Online Research 1, Fig. 2).


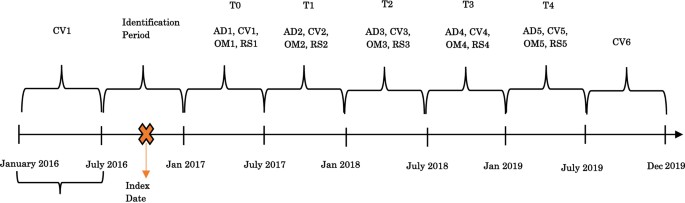


**Online Research 1, Fig. 2.** Study design by Paranjpe et al. T; time period; AD: adherence; CV; cardiovascular outcomes, OM; total other medications, RS; risk score. Source: Paranjpe et. al. [5]

The main limitations of this method are:

1. As mentioned above, this method only included patients with at least 1 month of overlap in the identification period with the attempt to include patients who were on combination therapy and, therefore, follow them up through electronic healthcare record (EHR). However, as shown in Online Research 1, Fig. 3, 30 days can be obtained in different ways, such as 30 consecutive days of co-exposure (i.e., Online Research 1, Fig. 3 - Case 1) or the sum of shorter periods of co-exposure to combination therapy (i.e., Online Research 1, Fig. 3 - Case 2). By using this definition, it is plausible that individuals who are no longer on combination therapy are included in the study (i.e., Online Research 1, Fig. 3 - Case 1), and those who start combination therapy at the end of the identification period are not included in the study (i.e., Fig. 3 - Case 3).
2. There is also a problem with transparency with the authors’ definition of overlap as it is not specified how 30 days of overlap are computed. If the authors meant only 30 consecutive days of co- exposure, this method will exclude patients such case 2 in Online Research 1, Fig. 3 which started, stopped, and re-started the combination therapy but still had a cumulative overlap of 30 days.


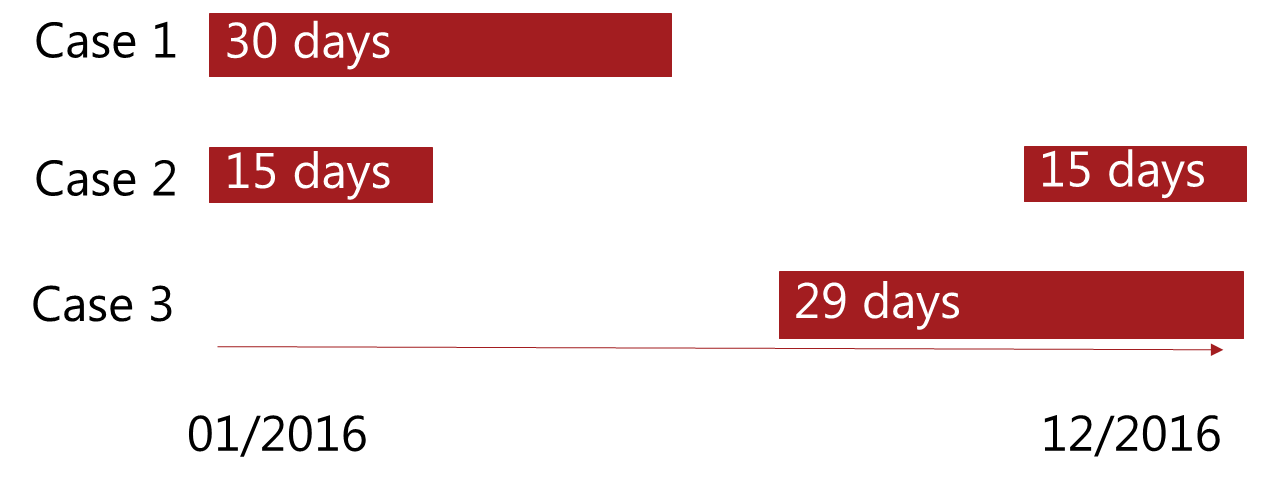


**Online Research 1, Fig. 3.** Two different pattern of 30 days co-exposure overlap (case 1: 30 days consecutive overlap and case 2: sum of shorter periods) and one case of late initiation of co-exposure.

1. Finally, in this method, patients are considered to be continuously exposed to combination therapy if they had at least two prescriptions, one in the identification period, one during the follow-up (i.e., Fig. 4 - Case 4). This is an unrealistic assumption as patients may start, stop, and re-start the combination therapy and, therefore, only assessing two prescriptions to define continuous exposure to combination therapy may lead to misclassification.


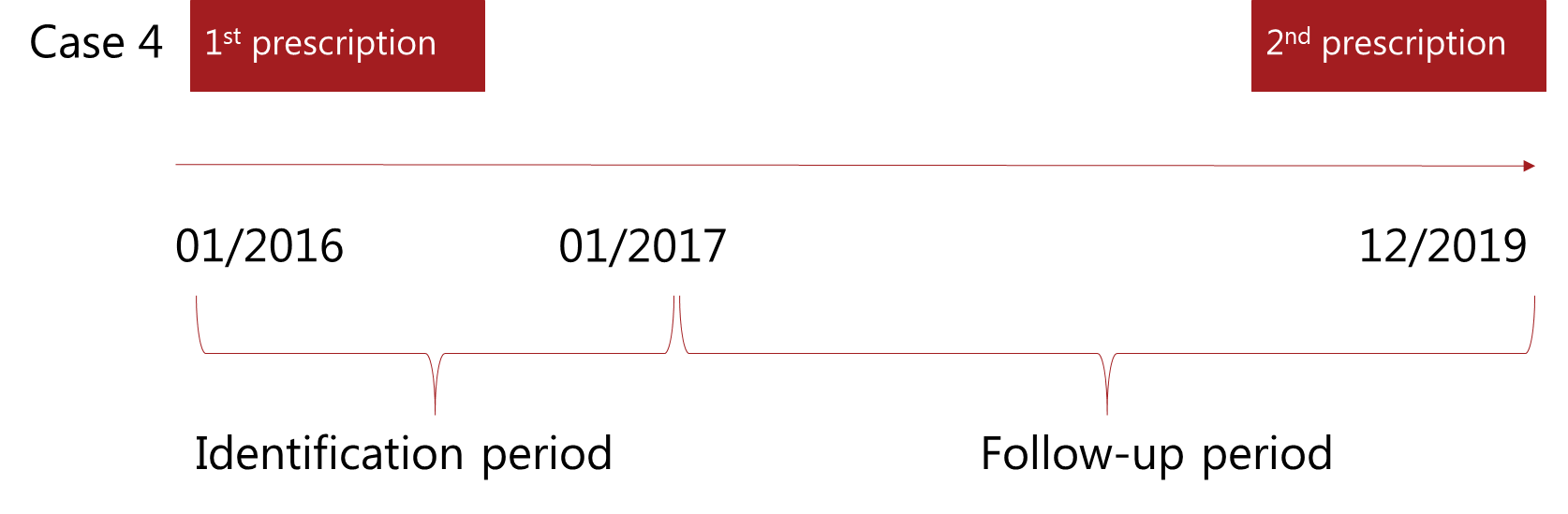


**Online Research 1, Fig. 4.** Pattern of one prescription in identification period and one prescription in follow-up period with a large gap in between.

**Article 2: Treciokiene et al., 2022**

In the study by Treciokiene et al., the authors used a method called the anniversary method to determine non-persistence to antihypertensive drug therapy [6]. Persistence is defined as *“the duration of time from initiation to discontinuation of therapy”* [17], and the measure can be expressed as the time until discontinuation of drug therapy. It is usually defined as a gap exceeding a predefined threshold between prescription claims. The simplest method of measuring persistence is the anniversary model. This model determines a patient to be persistent if a prescription is refilled within a specific interval surrounding the anniversary of the patient’s first prescription. The interval is determined by the researcher (e.g., anniversary day ± 30 days) [17].

Patients were included in the cohort if they were newly initiated with an antihypertensive in 2018, and the year 2017 was used to exclude prevalent users [6]. Combination therapy was defined as *“one medicine with more than one active pharmaceutical ingredients (API) or two or more medicines dispensed.”* In this method, the authors determined the index date as the first dispensing in 2018, and they determined the anniversary day as the last dispensing within 365 days from the index date [6]. Specifically, the last dispensation should have occurred 2 times the supply of the last dispensation (i.e., permissible gap) before the anniversary day. In this method, the authors used persistence as a surrogate of adherence. Patients are considered persistent if they received a dispensing within the permissible gap. A graphical display of the method is shown in Online Research 1, Fig. 5, where patient A is considered non-persistent, and patient B is considered persistent.


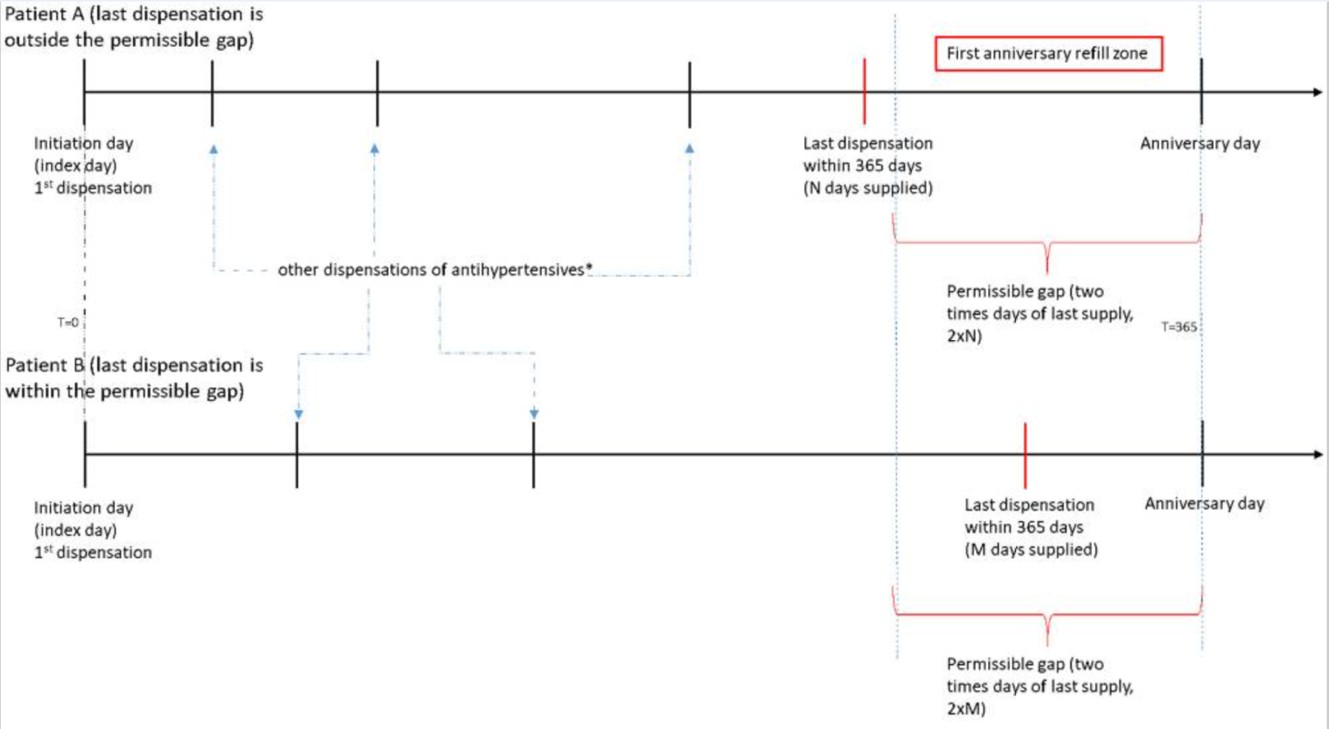


**Online Research 1, Fig. 5.** Study design by Treciokiene et al. Patient A: non-persistent and Patient B: persistent with the anniversary method. Source: Treciokiene et al. [6]

The main limitations of this method are:

1. By determining persistence solely based on the index date and the anniversary day, the prescription refills between these two prescriptions are not considered, and patients can be classified incorrectly as persistent in scenarios where patients might stop and restart treatment (e.g., for example due to adverse drug reactions) [18].
2. One limitation of using a permissible gap is that if the supply of the last dispensing is long, the permissible gap will cover a large proportion of the follow-up period, and this will classify patient as persistent even if they received only two prescriptions of which the last in the middle of the follow-up period. An example of this is given in Online Research 1, Fig. 6, where the last dispensing before the anniversary day is dispensed around the middle of the follow-up period. The days' supply of this last dispensing is 90 days, and therefore the permissible gap is 180 days, corresponding to nearly half of the follow-up time (i.e., 365 days).
3. The authors did not describe how they assessed the duration of medication events, compromising the transparency of the defined gap and the reproducibility of the study.


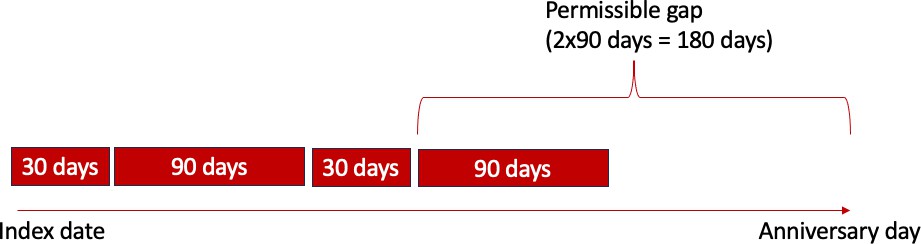


**Online Research 1, Fig. 6.** Example of a gap covering half the follow-up period when permissible gap is defined as 2 times the supply of the last dispensation.

**Article 3: Jeong et al., 2020**

In the article by Jeong et al. [7], Korean claims data from January 2011 to December 2015 were used to identify patients newly diagnosed with hypertension. The authors introduced a washout period of 12 months to exclude prevalent users, and the index date was defined as *“the first date of simultaneous diagnostic code for hypertension and prescription of antihypertensive drugs”*. Patients were followed for 365 days, and the follow-up period was divided into two periods: period 1 (day 1-180) and period 2 (day 181-365). Changes in treatment between the two periods were conceptually defined by the authors, as shown in Online Research 1, Fig. 7, along with 13 examples of case scenarios. The authors used persistence as a surrogate for adherence. Patients were considered persistent if they were exposed to any class of drugs between period 1 and period 2 with one of the patterns depicted in Online Research 1, Fig. 7 (except discontinuation). Furthermore, the authors used cumulative medication adherence (not specified) and defined *“good adherence as cumulative medication adherence of 80% or greater”* [7].


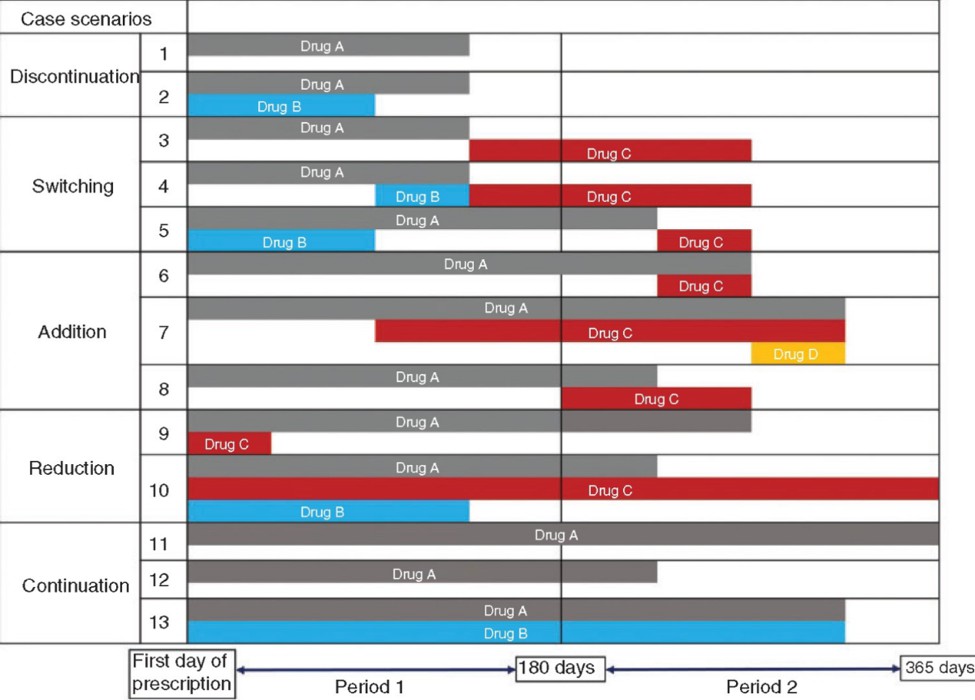


**Online Research 1, Fig. 7.** Definition of discontinuation and persistence (i.e., switching, addition, reduction, and continuation) in the study by Jeong et al. Source: Jeong et al. [7]

The main limitations of the study are:

1. There is no operative definition of discontinuation, switching, add-on, etc. Specifically, it is not explained how many days of co-exposure are necessary for each of the drugs or the specific algorithm for using this information to differentiate between switching, add-on, etc. Therefore, this method is not reproducible.
2. As mentioned, the authors considered patients as persistent if they continued any class(es) of drug from period 1 to period 2. This included patients who had a reduction of drugs from period 1 to period 2. According to the author, a reduction is considered a discontinuation of one of the drugs the patient was exposed to. Therefore, if a reduction occurred, it means the patient was not exposed to combination therapy. However, according to the author, he/she was persistent with combination therapy, which is not true (an example of this problem is provided in scenario 9 in Online Research 1, Fig. 7).
3. The authors did not explain how they calculated cumulative medication adherence. Consequently, the transparency of their methodology was compromised, making it impossible to replicate this study for investigating adherence to combination therapy. Additionally, it is not mentioned how they built the treatment episodes and how they assigned the duration of medication events.

**Article 4: Malo et al, 2021**

In the article by Malo et al. [8], new users of antihypertensives were recruited in 2015, using a 1-year washout period. From the index date (i.e., first prescription of antihypertensives in 2015) the authors assigned an index period of 15 days, during which patients were stratified into four cohorts based on their pharmacological treatments: *“monotherapy, fixed combination, free combination, and multiple mix”* as defined in section 3.3.4 [8]. Patients were followed through 2016, and persistence (used as surrogate for adherence) was evaluated using the gap method which has been defined by Pazzagli et al. as *“the gap method considers patients persistent if the time from the end of coverage of the redeemed prescription under investigation, and the date of redemption of the subsequent, is equal to or shorter than an allowed number of days (i.e., the permissible gap).”*[19]. Malo et al. defined the permissible gap as *“twice the length of the preceding prescription.”* The number of days of supply was estimated using the Defined Daily Dose (DDD) of the most recently dispensed prescription, thus defining the permissible gap as twice the number of DDD [8].

Patients were considered non-persistent if a prescription was outside the permissible gap during the follow-up. Furthermore, patients were categorized according to their patterns of use as: *“persistent, discontinuers, discontinuers who restarted, and spot-users (prescriptions only during the index period).”* The authors defined switching in the monotherapy and combination therapy cohorts as *“a switch from a single active principle to a different active principle or from a fixed combination to a different fixed combination or a free combination.”* Add-on was defined as *“the addition of a separate drug to the single active principle or the fixed combination initially prescribed and a change from a single active principle to a fixed combination in which one of the constituent drugs belonged to the same therapeutic subgroup was also considered add-on.”* [8]. A graphical representation of such pattern is presented in section 3.3.4, Fig. 9. Persistence in the monotherapy and fixed combination cohorts was assessed as *“class persistence (persistence with a medication from the same antihypertensive drug class, where a drug class was defined based on the 4th level of the ATC-code)”*[8], and persistence in the free combination cohort was assessed as *“therapy persistence (persistence with any antihypertensive medication).”* Additionally, the authors stated that *“the multiple mix cohort were not included in the persistence analysis.”*[8].

The main limitations of the study are:

1. The authors did not define how they assessed the cohorts of monotherapy, fixed combination, free combination, and multiple mix. Furthermore, they provided no definition for categorizing persistence, discontinuers, discontinuers who restarted, and spot-users. Therefore, the study lacks transparency, making it impossible to reproduce.
2. As mentioned, the authors stratified patients into four cohorts (monotherapy, fixed combination, free combination, and multiple mix) based on their type of treatment during the index period. By selecting a short index period of 15 days, the authors risk misclassifying patients into the wrong cohorts. Examples of this misclassification are provided in Online Research 1, Fig. 8. In Case 1, a patient is initially identified as a new user of drug 1 with a single active principle. During the index period, the patient is not starting a new therapy and is thus classified as belonging into the monotherapy cohort. However, this patient initiates therapy with drug 2, containing a different single active principle shortly after the end of the index period, which should classify the therapy as a free combination. The patient is, therefore, misclassified. In Case 2, a patient is initiated on monotherapy during the index period but is switched to a fixed combination during the follow-up. This patient would be categorized into the monotherapy cohort, which does not accurately reflect the type of treatment throughout the entire follow-up. In general, classifying patients during an index period without considering the entire follow-up period risks placing patients in the wrong cohorts.


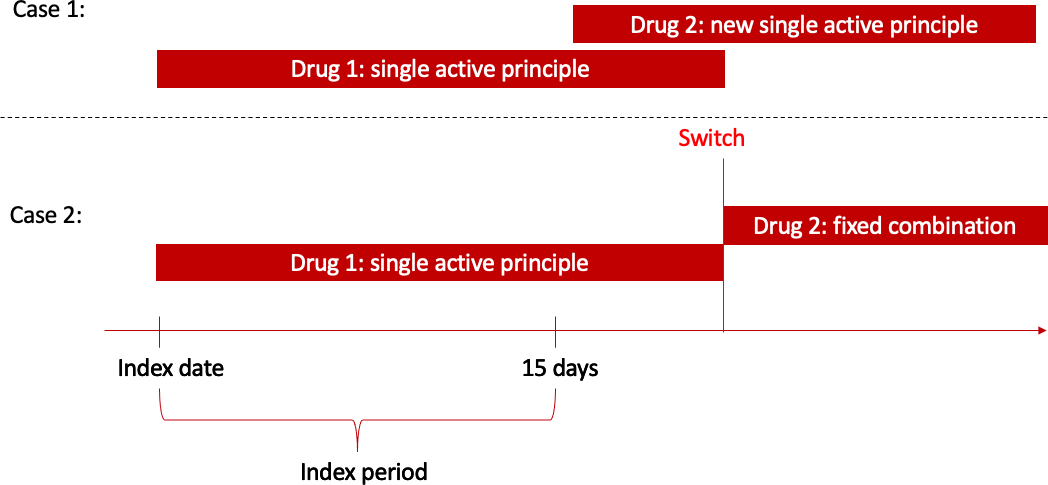


**Online Research 1, Fig. 8.** Classification of cohort during 15 days index period. In both case 1 and 2 the patients are misclassified in monotherapy cohort.

**Article 5: Rea et al., 2020**

In the article by Rea et al. [9], patients were recruited if they received a prescription for one or more antihypertensives during 2010. The first prescription was defined as the index date, and a washout period of 10 years was introduced to exclude prevalent users. Patients were followed for 1 year from the index date. Furthermore, patients were excluded based on the following exclusion criteria:

*“1) If they were prescribed a free dose combination therapy initially (i.e., two separate drugs).*

- 1. *If they did not renew any antihypertensive drug after the index prescription*
  2. *If they did not reach the 1-year follow-up (e.g., due to emigration or death)”* [9].

The duration of prescriptions was calculated using the DDD, and if stockpiling (i.e., redemption of new prescription before exhausting the available supply from last redemption) occurred, it was assumed that patients had used the entire amount of drug dispensed in the former prescription before starting a new one. Adherence was assessed using the PDC, where the ratio was defined as "*the number of days in which one or more antihypertensive drugs were available (irrespective of whether the drug or drugs were partly or totally different from the initial drugs initially prescribed) and the days of follow-up (i.e., 365 days)"* [9]. Patients were considered adherent if PDC was > 75%.

The main limitations of the study are:

1) When using retrospective data in pharmacoepidemiologic studies, excluding based on future events can introduce bias. In this study design, immortal time bias (section 3.3.2, Fig. 5) was introduced because the authors chose to exclude patients who did not renew their prescriptions after the index prescription. At some point, these patients might renew their prescription, and during the time between the index prescription and the renewal of the prescription, these patients are considered "immortal." This poses a problem for the study design because this group of patients would not be accurately categorized as patients who did not renew their prescriptions, as they eventually will renew them. The induced bias will affect the incidence rate of patients who did not renew their prescriptions.

**Article 6: Tajeu et al., 2019**

In the article by Tajeu et al. [10], new users of antihypertensive medications between 2007 and 2014 were identified in a United States (US) prescription claims database, with a washout period of 365 days to ensure that patients had no previous fills of antihypertensive medications. Prescriptions claims of antihypertensive medications included *“the date of the fill, drug class, days of supply obtained, and copay per day of supply.”* The follow-up period was 365 days from the index fill. The authors identified the patients' treatment regimen within seven days of the index prescription and categorized the regimen as either *“a single class, multiclass/multiple pill, or multiclass/combination therapy (combination therapy was defined as a single pill containing 2 or more antihypertensive classes)”* [10]. Adherence was calculated using interval-based PDC, and in the case of two or more classes of antihypertensive medications being dispensed, the numerator was defined as *“the number of days for which any antihypertensive medication was available during the follow-up period (i.e., the denominator).”* As depicted in Online Research 1, Fig. 9, the numerator was from A to D. Low adherence was defined as PDC < 80% [10].


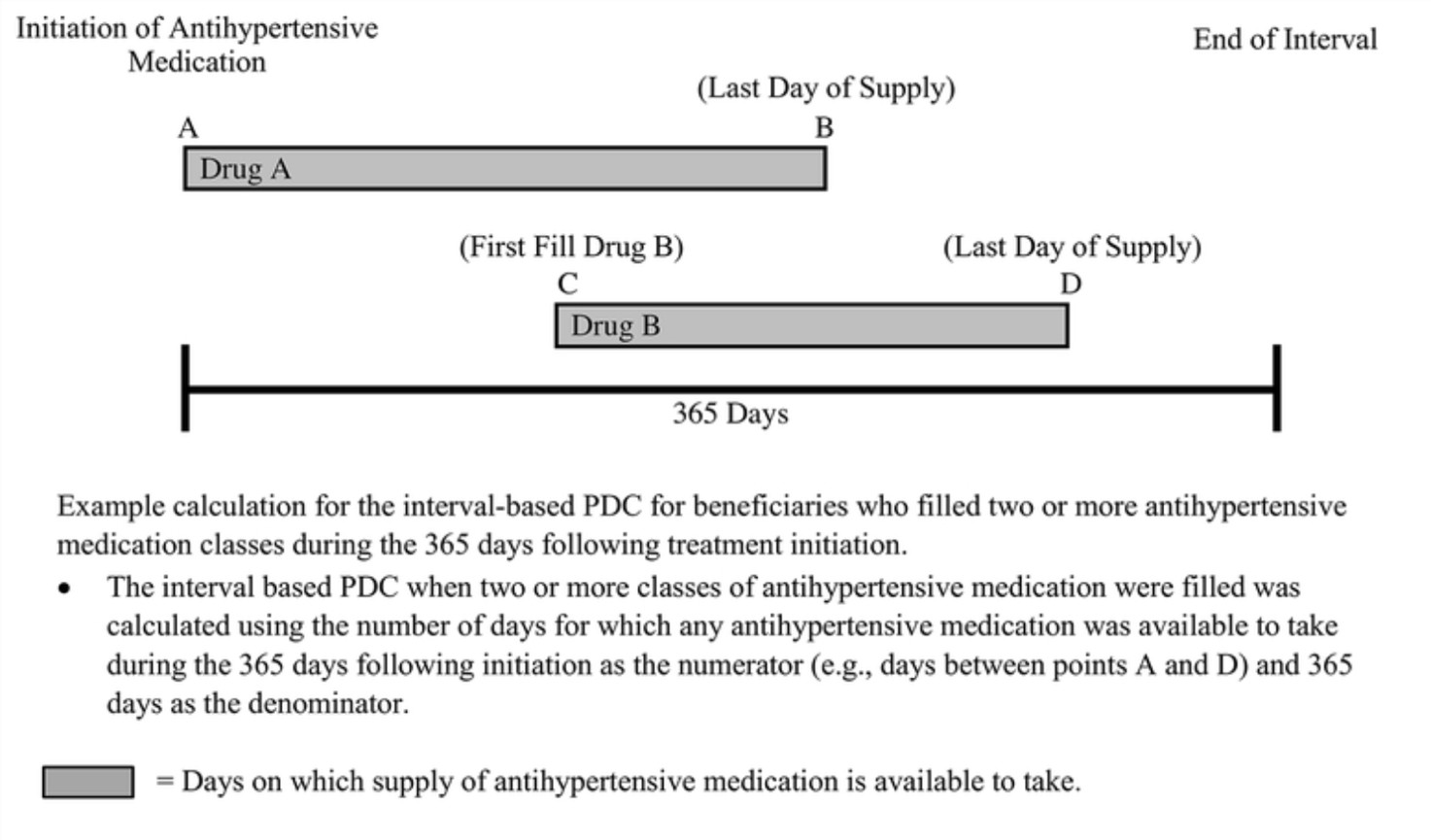


**Online Research 1, Fig. 9.** Study design using interval-based proportion of days covered (PDC) by Tajeu et al. Fig. from: Tajeu et al. [10]

The main limitations of the study are:

1. In the study, the authors assessed adherence to free combination therapy. However, they did not explain if or how they differentiated between switches and add-ons of different drug classes. When investigating adherence to free combination therapies, it is crucial to be able to differentiate between switches and add-ons because the wrong assessment of these factors can lead to incorrect data input in the calculation of PDC and, consequently, an inaccurate measurement of adherence.
2. As mentioned, the numerator in the calculation of PDC for free combination therapy was the number of days for which any antihypertensive medication was available during the follow-up period. A limitation of doing so is that adherence is likely to be overestimated. If a patient has only one drug of the combination therapy available during the entire follow-up period, this patient will have a PDC of 100% and be considered adherent. This example is depicted in Online Research 1, Fig. 10. However, despite drug 1 being available during the entire period, drug 2 is not, and therefore the patient will be wrongly classified as adherent to the combination therapy.


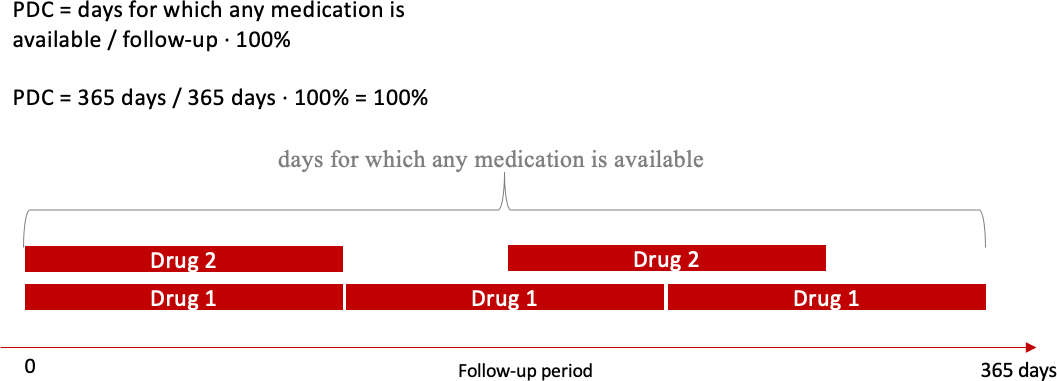
**Online Research 1, Fig. 10.** Overestimation of adherence to combination therapy when the numerator in proportion of days covered (PDC) is defined as: *“number of days for which any antihypertensive medication was available”* [10].

**Article 7: Holmqvist et al., 2018**

In the article by Holmqvist et al. [11], patients who dispensed three or more antihypertensives during 2006-2008 were recruited for the study. The index date was defined as *“the last registered blood pressure measurement taken after the 1st of July 2006.”* Eligibility for the study required that patients had a mean PDC ≥80% for at least three dispensed antihypertensive drugs in an index period of 180 days prior to the index date. Pharmacy refill information was obtained from a prescribed drug register. From the index date to day 365 and to day 730, adherence was measured through PDC, and to be classified as adherent *“a continuous PDC ≥80% for 3 antihypertensive drug classes was required. Additionally, switching within and between drug classes was allowed and stockpiling of antihypertensive drugs before index blood pressure were saved and accounted for”* [11]. An illustration of the study design is provided in Online Research 1, Fig. 11.


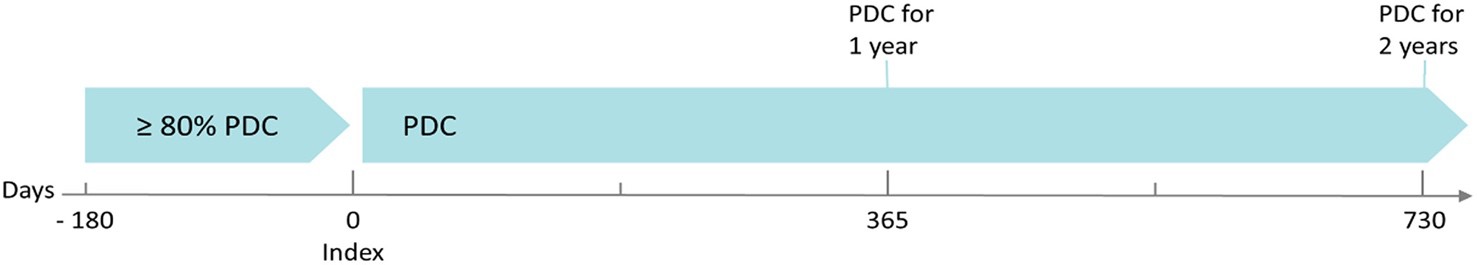


**Online Research 1, Fig. 11.** Study design by Holmqvist et al. PDC: Proportion of Days Covered. Source: Holmqvist et al. [11]

The main limitations of the study are:

1. By narrowing the cohort to patients who were already adherent, this study does not depict the general population.
2. The authors did not describe whether the duration of supply of the prescriptions was included in the prescribed drug register or if the authors made an estimation of the durations. Estimating the length of a prescription is a general methodological challenge in adherence research, and it is crucial to provide information on how the duration has been assessed [18]. Therefore, the transparency of the study was compromised, and it is not possible to reproduce the study.

3) The authors did not consider the introduction of treatment late in the observational window in the study design. In Online Research 1, Fig. 12, an example of calculating PDC in the first period from the index date to day 365 is given. The denominator will be 365 days, and the numerator will be the sum of days with available supply in the same period. In the example in Online Research 1, Fig. 12, drug 1 is initiated at the index date, and the supply is available during the entire observational window, resulting in a PDC of 100% (365 days/365 days · 100 %). However, if a patient starts treatment late in the observational window, as in the case of drug 2 and drug 3 given in Online Research 1, Fig. 12 at day 180 and day 270, respectively, the calculated PDCs will be 50% and 26%, respectively. Thus, the adherence measure will be influenced.


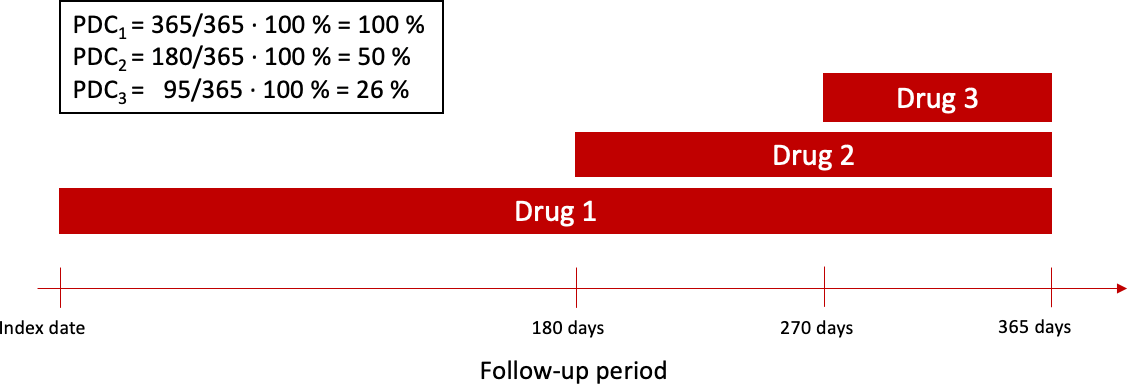


**Online Research 1, Fig. 12.** Examples of calculation of the Proportion of Days Covered (PDC) when drugs are introduced at three different time points during the follow-up period.

**Article 8: Ho et al., 2017**

In the article by Ho et al. [12], patients with an International Classification of Disease (ICD)-9 code of hypertension were identified in a national health insurance database from in the period January to December in 2009, and a washout period of 1 year was introduced to exclude prevalent patients. Inclusion criteria for the study included taking ≥1 antihypertensive medication, and the index date was defined as *“the first date on which medications were prescribed for hypertension”.* Patients were followed up for 1 year after the index date, and the authors collected data on the number of medications, the number of prescriptions, and the cumulative days of prescription for each type of antihypertensive medication [12].

Adherence to antihypertensive medications was assessed using medication possession ratio (MPR), defined as *“the total days of antihypertensive drugs supplied divided by the follow-up period (i.e., 365 days).”* For treatments with ≥1 type of antihypertensive medication, MPR was calculated for each type, and an average MPR was obtained as the overall MPR. The authors decided to exclude patients with a MPR <10% for any antihypertensive drugs. Patients were categorized into three groups according to their adherence: *“high adherence (MPR≥80%), medium adherence (50%<MPR<80%), and low adherence (MPR<50%) ”* [12].

The main limitations of the study are:

1. Using an overall MPR can present an inaccurate picture of the patients' medication patterns. An example is provided in Online Research 1, Fig. 13. In this example, the patient had an available supply of both drug 1 and drug 2 throughout the entire follow-up period, resulting in an MPR of 100% for both drug classes. However, towards the end of the follow-up period, drug 3 is added to the treatment regimen as an add-on. Since drug 3 was introduced late in the follow-up period, the available supply was relatively low compared to the 365-day follow-up period. Despite the patient being adherent to drug 3 from the initiation of drug 3 to the end of the follow-up period, the MPR for drug 3 would only be 12% (45 days/365 days · 100%). When averaging the MPRs of the three drug classes, the patient would achieve an overall MPR of 71%, which does not accurately reflect the patient's medication pattern and incorrectly categorizes the patient as having medium adherence (50%<MPR<80%). The correct categorization for this patient should be high adherence (MPR≥80%).


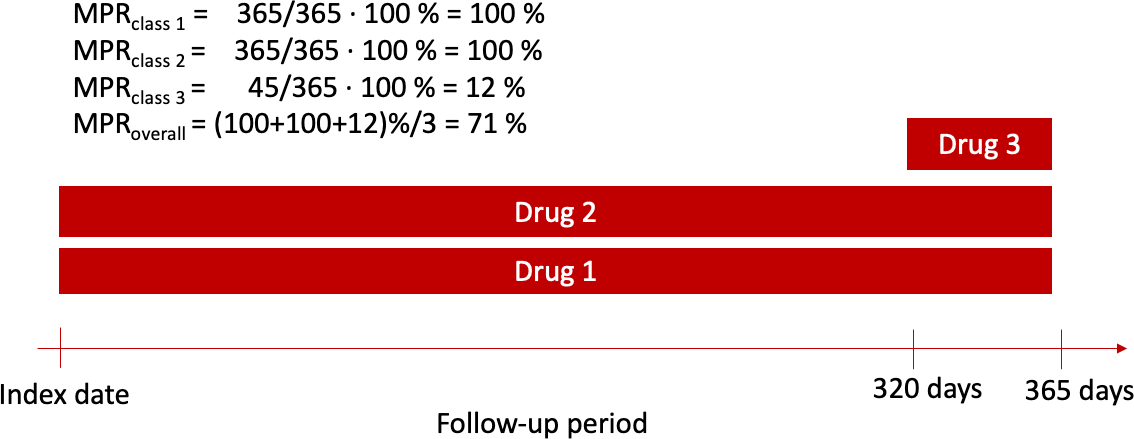


**Online Research 1, Fig. 13.** Example of calculation of an overall Medication Possession Ratio (MPR) when two drugs are introduced at index date and one drug is introduced late in the follow-up period.

1. As mentioned, the authors decided to exclude patients with a MPR <10%. However, by excluding these patients, the authors introduce selection bias in the form of immortal time bias (section 3.3.2, Fig. 5). Furthermore, they introduce the healthy adherer (section 3.3.2) effect as they only include patients with higher adherence.

**Article 9: Yang et al., 2017**

In the article by Yang et al. [13], patients were recruited during 2007-2008 if they met the defined criteria, which, among other requirements, included having had no antihypertensive medications for the 12 months before the date of their first hypertension diagnosis (ICD-9) and at least one prescription fill of an antihypertensive after the first hypertension diagnosis [13]. The authors determined the patients' follow-up in months, and it was measured from *“the index prescription date to the first occurrence of a CVD event, death, or the end of the follow-up period (December 31, 2014)”.* Using the prescription fill date and the days' supply, they counted the number of days covered by at least one antihypertensive medication. Stockpiling of prescriptions for the same medication were adjusted to start on the day the previous supply ended. Adherence was defined as *“the interval-based cumulative PDC of ≥1 antihypertensives during the follow-up.”* The authors used PDC both as a categorical variable, defining it as *“low adherence (PDC < 40%), intermediate adherence (PDC = 40–79%), and high adherence (PDC ≥ 80%), and as a continuous variable”* [13].

The main limitation of the study is:

1. The authors defined adherence as *“the interval-based cumulative PDC of ≥1 antihypertensives during the follow-up”* [13]. When using such definition, the adherence will reflect a monotherapeutic treatment regimen. That is because Drug A will define the number of days of drug supply as seen in Online Research 1, Fig. 14. Therefore, both Drug B and Drug C will not influence the PDC. Additionally, the authors did not define how to differentiate between add-ons and switches in a combination therapy regimen in their study design.


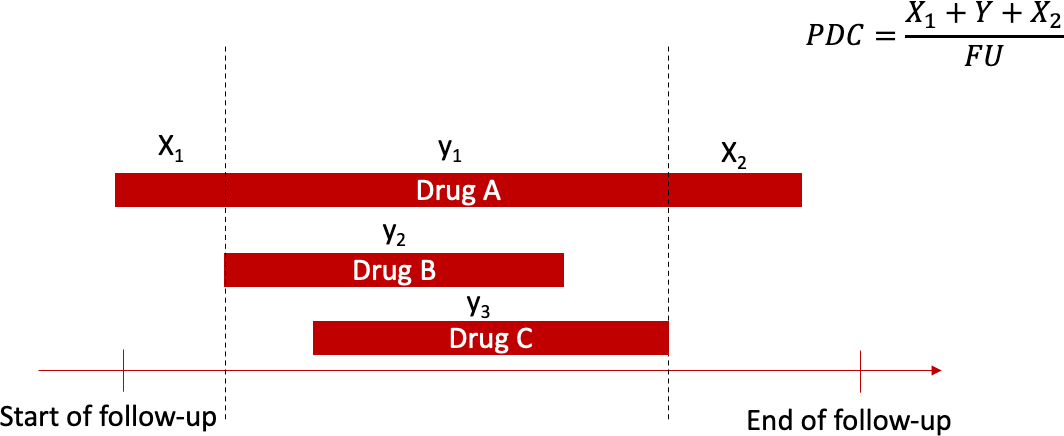


**Online Research 1, Fig. 14.** Illustration of the interval-based cumulative Proportion of Days Covered (PDC).

**Article 10: Tang et al., 2017**

In the article by Tang et al. [14], the study population consisted of patients with incident hypertension, which was defined as *“one hospitalization or two physician claims within 2 years with a hypertension ICD code between April 1, 2004, and March 31, 2005.”* The index date was defined as the first date when the criteria for incident hypertension were fulfilled, and the diagnosis incidence was determined with a 3-year washout period. The authors utilized a prescription database with drug data covering the period from April 1, 2002, to March 31, 2009. However, the authors did not mention whether the duration of prescriptions was estimated or if this information was included in the drug data. The authors decided to exclude patients who *“did not have at least a single prescription refill within one year after the first prescription fill.”* Furthermore, the authors stated: *“for patients on antihypertensive therapy prior to the index date, adherence measures were calculated from the first prescription fill after April 1, 2002 (i.e., the start of the period covered in the prescription registry).”*

The authors aimed to *“compare adherence rates using different operational definitions: PDC, MPR interval-based, and MPR prescription-based”* [14]. The following formulas below were used:

$$PDC=\frac{number of days where at least one medication was available}{365 days}$$

$$MPR_{interval-based}=\frac{Days supply of medication}{365 days}, capped at 1$$

$$MPR_{prescription-based}=\frac{Days supply of medication excluding supply from last fill}{Last refill date-First refill date}, capped at 1$$

For patients on polytherapy, four different MPRs (both interval-based and prescription-based) were calculated [14]. The definitions of these are shown in Online Research 1, Table 3. Both MPRs and PDC were dichotomized, and the authors used a threshold of 80% to indicate good adherence.


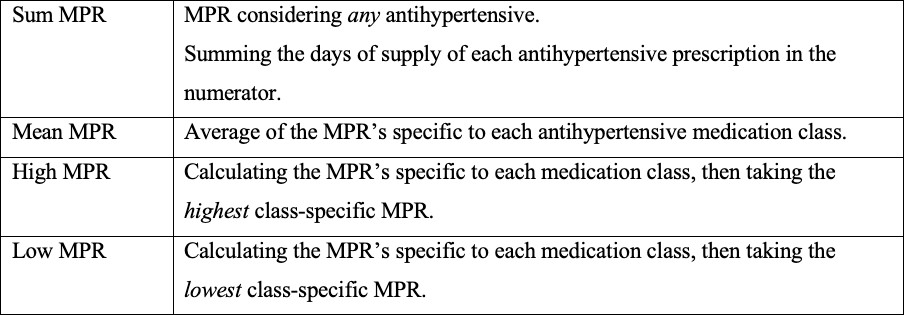
**Online Research 1, Table 3.** Four different Medication Possession Ratio (MPR) interval-based and prescription-based. Source: Tang et al. [14]

The main limitations of the study are:

1. As mentioned, the authors were not transparent about how the duration of prescriptions was estimated, compromising the reproducibility of the study.
2. By excluding patients without at least a second refill of antihypertensive medications, the authors introduced immortal time bias (section 3.3.2, Fig. 5).
3. The authors mixed incident and prevalent users of antihypertensives, thereby introducing the healthy adherer effect (section 3.3.2).
4. Despite the authors giving thought to different operational definitions, the adherence measures have limitations and problems. The authors did not explain if they considered how to differentiate between add-ons and switches. For the MPR measures, the sum MPR is likely to overestimate adherence, and as in the study by Ho et al. [12], taking the mean MPR is not useful as it cannot provide information about the patient's medication patterns. Similarly, simply taking the highest or lowest MPR of the medication classes is not useful either.

**Article 11: Levi et. al., 2016**


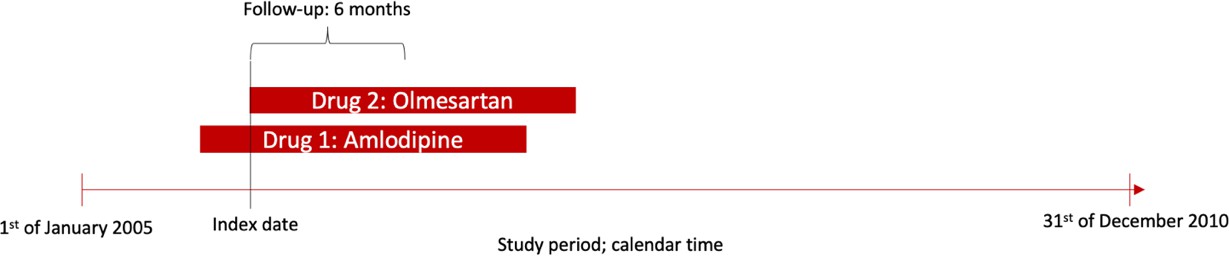
In the article by Levi et al. [15], the objective was *“to compare adherence with Olmesartan/Amlodipine fixed-dose combination (FDC) and the extemporaneous combination (i.e., in this study the free dose combination)”* [15]. The two study cohorts consisted of patients diagnosed with hypertension, as indicated by ICD-9 codes, and using either of the mentioned treatment regimens starting from the time when the two different formulations became available in Italy: January 1, 2005, for the extemporaneous combination users, and September 1, 2011, for the FDC users. For the extemporaneous combination users, an overlapping prescription were defined as *“a prescription for 1 of the 2 components before or on the run-out date of the other component.”* The index date for extemporaneous combination users was defined as the first prescription date of the second component overlapping with the first component. The study design for extemporaneous combinations is illustrated in Online Research 1, Fig. 15. Furthermore, the authors stated: *“patients were considered eligible if they had 6 months of follow-up. Patients were followed from the index date until the end of data coverage, the end of the study period (December 31, 2010, for the cohort of extemporaneous combinations and December 31, 2012, for the cohort with fixed-dose combinations), the last day of the sixth month of follow-up, or death, whichever occurred first”* [15].

**Online Research 1, Fig. 15.** Study design by Levi et al. for free dose combinations.

The duration of each prescription was calculated using the defined daily dose, and adherence was estimated by calculating the PDC. Patients were classified into three adherence levels according to the PDC: “*high (PDC ≥ 80%), intermediate (PDC = 40%-79%), or low (PDC < 40%)”* [15].

The main limitation of the study is:

1) As mentioned, the index date for extemporaneous-combination users was defined as the first prescription date of the second component overlapping with the first component. As shown in Online Research 1, Fig. 15, by choosing the index date as the date of the second component, the authors are conditioning on future events, thereby introducing immortal time bias (section 3.3.2, Fig. 5).

**Article 12: Machnicki et al., 2015**

In the article by Machnicki et al. [16], the aim was *“to determine whether the Amlodipine/Valsartan/Hydrochlorothiazide single-pill combination (SPC) is associated with improved adherence compared to the corresponding free-dose combination (FDC)”* [16]. The study population included adult patients diagnosed with hypertension according to ICD-9 codes between October 2009 and December 2011. For the FDC cohort *“at least 15 days of concurrent use (overlapping days supplied) of separate prescriptions for amlodipine, valsartan, and hydrochlorothiazide”* were required. The index date in the FDC cohort was the last fill date when the combination therapy was filled on different dates. The study design included a 12-month pre- index-date baseline period. If patients had prescription claims for the same combination within this period (different SPCs, components of the SPC, and FDC monotherapies), they were still included in the cohort. Patients were followed for 12 months after the index date, and adherence was measured with PDC and MPR. They calculated the PDC for the index drug and found the proportion of patients with a PDC≥80%. For FDCs *“MPR was calculated separately for each component, and the lowest MPR was used as an indication of the FDC MPR.”* Likewise, MPR was dichotomized, and patients were considered adherent if MPR≥80% [16].

The main limitations of the study are:

1. As mentioned, the index date in the FDC cohort was the last fill date when the combination therapy was filled on different dates. By choosing the date of the last prescription as the index date, the authors were conditioning on future events and introducing immortal time bias (section 3.3.2, Fig. 5), as observed in the study by Levi et al. [15].
2. As mentioned, the authors did not employ a new user design and included prevalent users in the study cohort. By doing so, they mixed incident and prevalent users, introducing the healthy adherer effect (section 3.3.2).
3. The authors calculated the MPR separately for each component of the FDCs, and the lowest MPR was used as the adherence measure. This approach is similar to the low MPR operational definition used in the study by Tang et al. [14], which is not a useful measure, as it does not reflect the patient's medication patterns.
4. Lastly, the transparency and reproducibility of the study are limited as the authors did not specify how the duration of prescriptions was estimated, nor did they explain how they differentiated between add-ons and switches.

**References**

1. Wood EH (1994) MEDLINE: the options for health professionals. Journal of the American Medical Informatics Association 1:372–380

2. Nieuwlaat R, Wilczynski N, Navarro T, et al (2014) Interventions for enhancing medication adherence. Cochrane database of systematic reviews

3. Arguedas JA, Leiva V, Wright JM (2020) Blood pressure targets in adults with hypertension. Cochrane Database of Systematic Reviews

4. Moher D, Liberati A, Tetzlaff J, et al (2009) Preferred reporting items for systematic reviews and meta-analyses: the PRISMA statement. PLoS Med 6:. https://doi.org/10.1371/JOURNAL.PMED.1000097

5. Paranjpe R, Johnson ML, Chen H, et al (2022) Evaluating Adherence to Concomitant Diabetes, Hypertension, and Hyperlipidemia Treatments and Cardiovascular Outcomes Among Elderly Patients Using Marginal Structural Modeling. High Blood Press Cardiovasc Prev 29:601–610. https://doi.org/10.1007/S40292-022-00543-4

6. Treciokiene I, Bratcikoviene N, Gulbinovic J, et al (2022) Non-persistence to antihypertensive drug therapy in Lithuania. Eur J Clin Pharmacol 78:1687. https://doi.org/10.1007/S00228-022-03369-0

7. Jeong SM, Kim S, Wook Shin D, et al (2021) Persistence and adherence to antihypertensive drugs in newly treated hypertensive patients according to initial prescription. Eur J Prev Cardiol 28:E1–E4. https://doi.org/10.1177/2047487319900326

8. Malo S, Aguilar-Palacio I, Feja C, et al (2021) Effect of patient and treatment factors on persistence with antihypertensive treatment: A population-based study. PLoS One 16:. https://doi.org/10.1371/JOURNAL.PONE.0245610

9. Rea F, Savaré L, Franchi M, et al (2021) Adherence to Treatment by Initial Antihypertensive Mono and Combination Therapies. Am J Hypertens 34:1083–1091. https://doi.org/10.1093/AJH/HPAB083

10. Tajeu GS, Kent ST, Huang L, et al (2019) Antihypertensive Medication Nonpersistence and Low Adherence for Adults <65 Years Initiating Treatment in 2007-2014. Hypertension 74:35–46. https://doi.org/10.1161/HYPERTENSIONAHA.118.12495

11. Holmqvist L, Boström KB, Kahan T, et al (2018) Drug adherence in treatment resistant and in controlled hypertension-Results from the Swedish Primary Care Cardiovascular Database (SPCCD). Pharmacoepidemiol Drug Saf 27:315–321. https://doi.org/10.1002/PDS.4388

12. Ho CP, Yeh JI, Wen SH, Lee TJF (2017) Associations among medication regimen complexity, medical specialty, and medication possession ratio in newly diagnosed hypertensive patients: A population-based study. Medicine 96:. https://doi.org/10.1097/MD.0000000000008497

13. Yang Q, Chang A, Ritchey MD, Loustalot F (2017) Antihypertensive Medication Adherence and Risk of Cardiovascular Disease Among Older Adults: A Population-Based Cohort Study. J Am Heart Assoc 6:. https://doi.org/10.1161/JAHA.117.006056

14. Tang KL, Quan H, Rabi DM (2017) Measuring medication adherence in patients with incident hypertension: a retrospective cohort study. BMC Health Serv Res 17:. https://doi.org/10.1186/S12913-017-2073-Y

15. Levi M, Pasqua A, Cricelli I, et al (2016) Patient Adherence to Olmesartan/Amlodipine Combinations: Fixed Versus Extemporaneous Combinations. J Manag Care Spec Pharm 22:255–262. https://doi.org/10.18553/JMCP.2016.22.3.255

16. Machnicki G, Ong SH, Chen W, et al (2015) Comparison of amlodipine/valsartan/hydrochlorothiazide single pill combination and free combination: adherence, persistence, healthcare utilization and costs. Curr Med Res Opin 31:2287–2296. https://doi.org/10.1185/03007995.2015.1098598

17. Caetano PA, Lam JMC, Morgan SG (2006) Toward a standard definition and measurement of persistence with drug therapy: Examples from research on statin and antihypertensive utilization. Clin Ther 28:1411–1424. https://doi.org/10.1016/J.CLINTHERA.2006.09.021

18. Pazzagli L, Liang D, Andersen M, et al (2022) Rationale and performances of a data-driven method for computing the duration of pharmacological prescriptions using secondary data sources. Sci Rep 12:6245. https://doi.org/10.1038/s41598-022-10144-9

19. Pazzagli L, Andersen M, Sessa M (2022) Pharmacological and epidemiological considerations while constructing treatment episodes using observational data: A simulation study. Pharmacoepidemiol Drug Saf 31:55–60. https://doi.org/10.1002/pds.5366

# Online Research 2

Maurizio Sessa

2024-06-26

##########################################################################
# A Newly Developed Method for Assessing Co-Exposure to Free Dose #
# Combinations: A Proof-of-Concept Study using Antihypertensive #
# Medications in Danish Registers #
# #
# Developer: Maurizio Sessa #
# Code v: 1 #
##########################################################################

# Load required libraries
library(dplyr)
library(AdhereR)
library(data.table)

data <- data.table(
 PatientID = c(2, 2, 2, 2, 2, 2, 3, 3, 3, 3, 3, 3, 3, 3, 3, 3, 3, 3, 3, 3, 3, 3, 3, 3, 3, 3, 3, 3, 3, 3, 3, 3, 3, 3, 3, 3, 3, 3, 3, 3, 3, 3, 3, 3, 3),
 Drug = c("C03CA01", "C09AA01", "C09AA01", "C09AA01", "C09AA01", "C09AA01",
 "C09AA02 ", "C09AA02 ", "C09AA02 ", "C09AA02 ", "C09AA02 ", "C09AA02",
 "C09AA02", "C09AA02", "C09AA02",
 "C09CA01", "C09CA01", "C09CA01", "C09CA01", "C09CA01", "C09CA01",
 "C02CA02 ", "C02CA02 ", "C02CA02 ",
 "C03AA01",
 "C03AA01", "C03AA01", "C03AA01", "C03CA01", "C03CA01", "C03CA01", "C03CA01", "C03CA01", "C03CA01", "C03CA01", "C03CA01",
 "C09AA02", "C09AA02", "C09AA02", "C09AA02", "C03CA01", "C03CA01", "C03CA01", "C03CA01", "C03CA01"),
 StartDate = as.Date(c("2057-04-14", "2056-10-21", "2057-04-14", "2057-06-24", "2058-02-08", "2058-05-31", "2056-10-18", "2056-12-26", "2057-03-18", "2057-06-25", "2057-09-02", "2056-10-18", "2057-03-18", "2057-06-25", "2057-09-02", "2057-09-02", "2056-10-18", "2057-01-28", "2057-05-02", "2057-07-28", "2057-10-17", "2057-01-28", "2057-05-02", "2057-09-02", "2056-10-18", "2056-10-18", "2056-12-26", "2057-10-17", "2056-12-26", "2057-03-18", "2057-06-25", "2057-09-02", "2056-10-18", "2057-01-28", "2057-05-02", "2057-07-28", "2057-02-04", "2057-07-09", "2057-05-20", "2057-09-02", "2057-09-02", "2056-10-18", "2057-05-02", "2056-12-30", "2057-03-18")),
 EndDate = as.Date(c("2057-07-13", "2056-12-20", "2057-06-13", "2057-08-23", "2058-04-09", "2058-07-30", "2056-11-24", "2057-01-25", "2057-04-17", "2057-07-25", "2057-10-02", "2056-11-17", "2057-04-17", "2057-07-20", "2057-10-02", "2057-12-16", "2056-11-17", "2057-02-27", "2057-05-17", "2057-08-27", "2057-11-16", "2057-02-27", "2057-06-01", "2057-10-02", "2056-11-29", "2056-12-02", "2057-03-26", "2057-11-14", "2057-01-25", "2057-04-17", "2057-07-15", "2057-10-02", "2056-11-17", "2057-02-27", "2057-06-01", "2057-08-27", "2057-03-04", "2057-08-04", "2057-06-17", "2057-12-31", "2057-10-02", "2056-11-17", "2057-06-01", "2057-01-29", "2057-04-17"))
)


# Convert date columns to Date objects
data$StartDate <- as.Date(data$StartDate)
data$EndDate <- as.Date(data$EndDate)

# Initialize an empty dataframe to store the output of the co-exposure function
output_df <- data.frame(PatientID = character(),
 DrugPairs = character(),
 StartCoExposure = as.Date(character()),
 EndCoExposure = as.Date(character()),
 NumDaysCoExposure = numeric(),
 stringsAsFactors = FALSE)

# The calculation of duration of co-exposure iterated over each patient (Step 2)
for (patient in unique(data$PatientID)) {
 patient_data <- data %>% filter(PatientID == patient)
 drugs <- unique(patient_data$Drug)

 # Check if the patient has at least 2 unique drugs
 if (length(drugs) < 2) {
 next
 }

 # Generate combinations of drugs
 drug_combinations <- combn(drugs, 2)

 # Iterate over each drug combination
 for (i in 1:ncol(drug_combinations)) {
 drug1 <- drug_combinations[1, i]
 drug2 <- drug_combinations[2, i]

 # Find all time points where both drugs are present
 common_time_points <- which(patient_data$Drug == drug1 | patient_data$Drug == drug2)

 # Iterate over each time point
 for (time_index1 in common_time_points) {
 for (time_index2 in common_time_points) {
 # Check if both drugs are present at this time point and calculate overlap
 if (time_index1 != time_index2 && patient_data$Drug[time_index1] == drug1 && patient_data$Drug[time_index2] == drug2) {
 start_date <- max(patient_data$StartDate[time_index1], patient_data$StartDate[time_index2])
 end_date <- min(patient_data$EndDate[time_index1], patient_data$EndDate[time_index2])
 if (start_date <= end_date) {
 num_days <- as.numeric(end_date - start_date + 1)

 # Add data to output dataframe
 output_df <- rbind(output_df, data.frame(PatientID = patient,
 DrugPairs = paste(drug1, drug2, sep = "-"),
 StartCoExposure = start_date,
 EndCoExposure = end_date,
 NumDaysCoExposure = num_days))
 }
 }
 }
 }
 }
}
# Reset row names of the output dataframe
rownames(output_df) <- NULL

# Print the output dataframe
print(output_df)

# Function to check for overlap
check_overlap <- function(start1, end1, start2, end2) {
 return(max(start1, start2) <= min(end1, end2))
}

# Function to find overlapping periods for each patient
find_overlaps <- function(df) {
 # Initialize an empty list to store results
 overlap_periods <- list()

 # Iterate over each unique PatientID
 for (patient in unique(df$PatientID)) {
 patient_data <- df %>% filter(PatientID == patient)
 num_drug_pairs <- nrow(patient_data)

 for (i in 1:(num_drug_pairs-1)) {
 for (j in (i+1):num_drug_pairs) {
 if (check_overlap(patient_data$StartCoExposure[i], patient_data$EndCoExposure[i],
 patient_data$StartCoExposure[j], patient_data$EndCoExposure[j])) {
 overlap_start <- max(patient_data$StartCoExposure[i], patient_data$StartCoExposure[j])
 overlap_end <- min(patient_data$EndCoExposure[i], patient_data$EndCoExposure[j])
 overlap_periods <- append(overlap_periods, list(
 data.frame(PatientID = patient,
 OverlapStart = overlap_start,
 OverlapEnd = overlap_end,
 Pair1 = patient_data$DrugPairs[i],
 Pair2 = patient_data$DrugPairs[j])
 ))
 }
 }
 }
 }
 return(do.call(rbind, overlap_periods))
}

# Find overlapping periods
overlapping_periods <- find_overlaps(output_df)
print(overlapping_periods)

# Function to combine pairs and sort alphabetically
combine_pairs <- function(pair1, pair2) {
 unique_drugs <- unique(unlist(strsplit(c(pair1, pair2), split = "-")))
 sorted_drugs <- sort(unique_drugs)
 return(paste(sorted_drugs, collapse = "-"))
}

# Apply the function to create a new column for combined drugs
overlapping_periods <- overlapping_periods %>%
 rowwise() %>%
 mutate(CombinedDrugs = combine_pairs(Pair1, Pair2)) %>%
 ungroup()

# Remove Pair1 and Pair2 columns if no longer needed
overlapping_periods <- overlapping_periods %>% select(-Pair1, -Pair2)
overlapping_periods <- unique(overlapping_periods)


#Medication events plotting
pt3a = data[which(data$PatientID==c("2","3")),]

pt3b = output_df[which(output_df$PatientID==c("2","3")),]
pt3c = overlapping_periods[which(overlapping_periods$PatientID==c("2","3")),]

pt3a$Duration = as.numeric(pt3a$EndDate-pt3a$StartDate)
medication_events <- CMA0(data=pt3a,
 ID.colname="PatientID",
 event.date.colname="StartDate",
 event.duration.colname="Duration",
 medication.class.colname="Drug",
 followup.window.start=0,
 observation.window.start=0,
 observation.window.duration=365*2,
 date.format="%m/%d/%Y")
plot(medication_events)


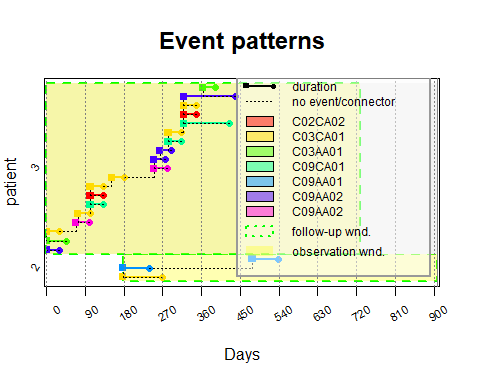


##########################
# END #
##########################

**Online Research 3. Validation of the co-exposure function**

The following section explains and simulates the R code to generate a plot that shows all medication events for all patients (patients 2 and 3 from the simulated data in Online Research 2). The code snippets are written on a grey background. The CMA0 from the AdhereR package [1] function was used to plot the medication events. In this simulated example the OW was set to 730 days. In the study it was set to reflect the initial period (day 180).

*#Medication events plotting*

medication_events <- **CMA0**(data=pt3a,

ID.colname="PatientID", event.date.colname="StartDate", event.duration.colname="Duration", medication.class.colname="Drug", followup.window.start=0, observation.window.start=0, observation.window.duration=365*2, date.format="%m/%d/%Y")

**plot**(medication_events)

The plot from the simulated data is presented below. As seen in the output from the co-exposure function in Online Research 2, both patients have co-exposure. In the following section how to assess the start and end of co-exposure for patients 2 and 3 in the plot will be described.


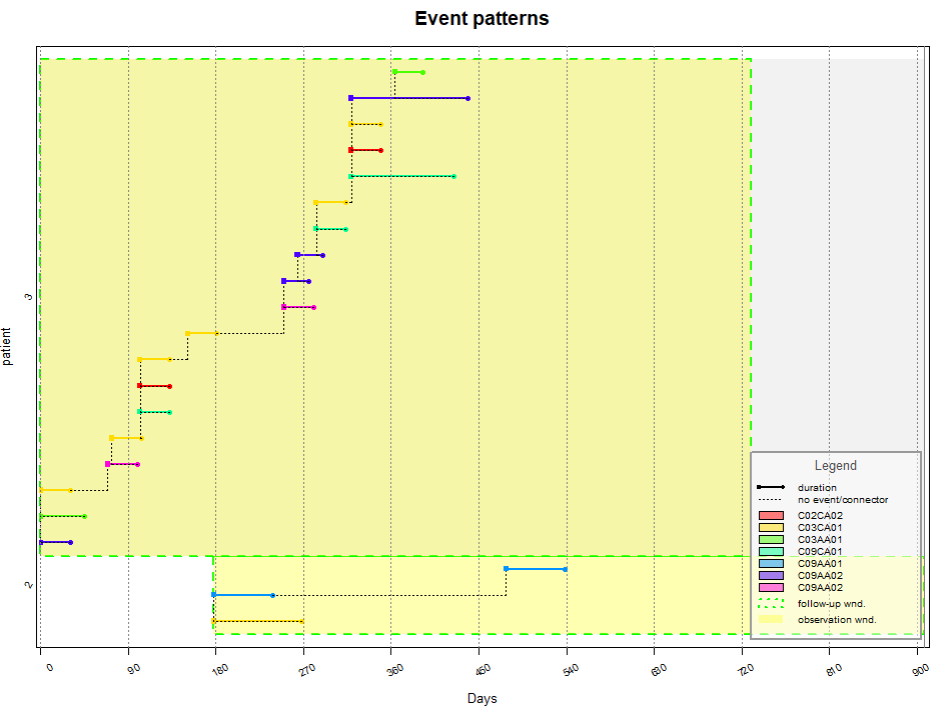


**Online Research 3, Fig. 1.** Event pattern of 2 hypothetical patients constructed with simulated data.

Start and end of co-exposure is determined at the first time point where medication events of two different drugs overlap, and as the last time point where medication events of two different drugs overlap, respectively. On the Fig. below the start and end of co-exposure is marked with black arrows for patient 2.


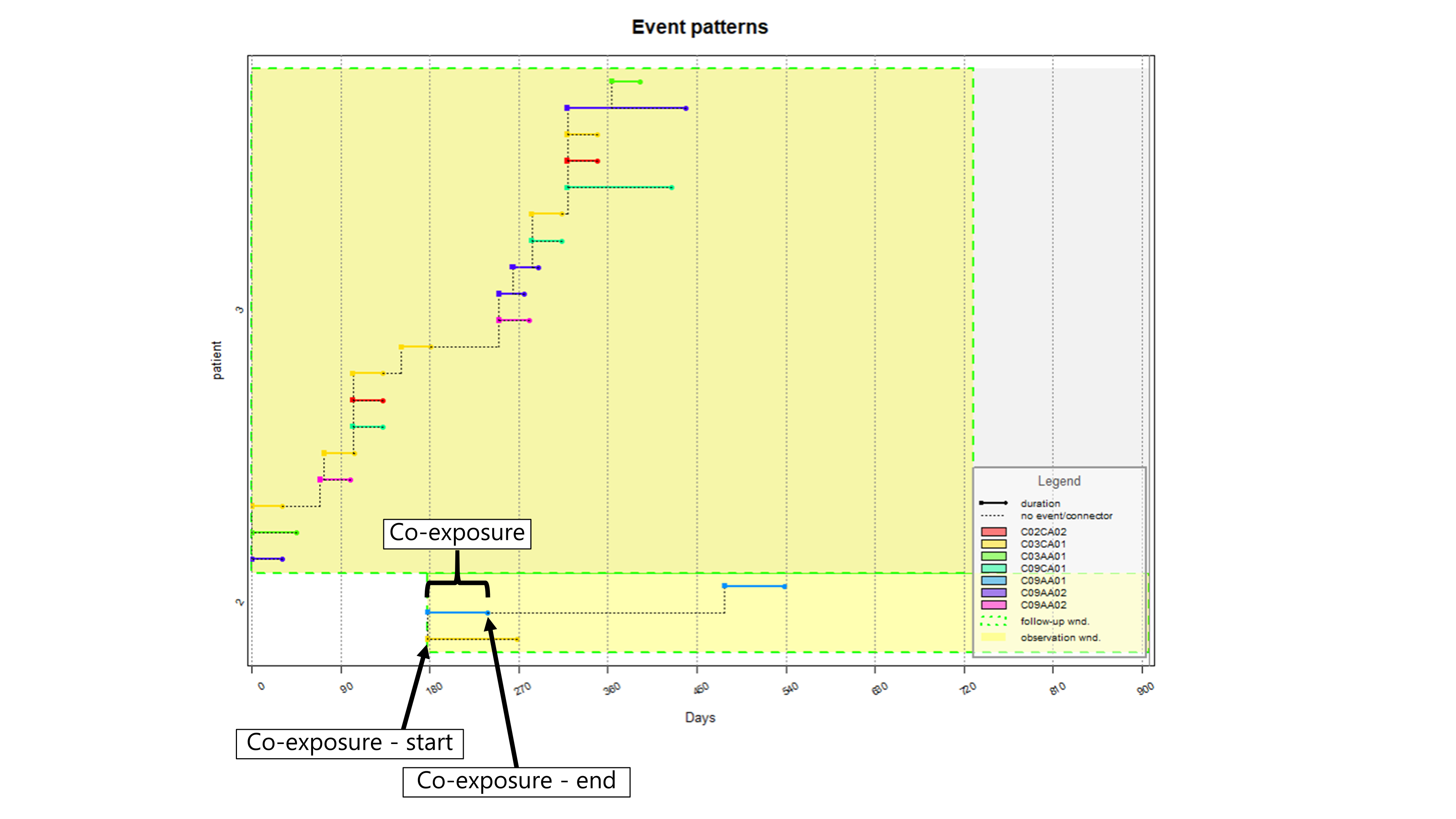


**Online Research 3, Fig. 2.** Event pattern of 2 hypothetical patients constructed with simulated data.

**References**

1. Dima AL, Dediu D (2017) Computation of adherence to medication and visualization of medication histories in R with AdhereR: Towards transparent and reproducible use of electronic healthcare data. PLoS One 12:. https://doi.org/10.1371/JOURNAL.PONE.0174426
